# A scalable, fully automated process for construction of sequence-ready human exome targeted capture libraries

**DOI:** 10.1186/gb-2011-12-1-r1

**Published:** 2011-01-04

**Authors:** Sheila Fisher, Andrew Barry, Justin Abreu, Brian Minie, Jillian Nolan, Toni M Delorey, Geneva Young, Timothy J Fennell, Alexander Allen, Lauren Ambrogio, Aaron M Berlin, Brendan Blumenstiel, Kristian Cibulskis, Dennis Friedrich, Ryan Johnson, Frank Juhn, Brian Reilly, Ramy Shammas, John Stalker, Sean M Sykes, Jon Thompson, John Walsh, Andrew Zimmer, Zac Zwirko, Stacey Gabriel, Robert Nicol, Chad Nusbaum

**Affiliations:** 1 Genome Sequencing Platform, Broad Institute of MIT and Harvard, 320 Charles Street, Cambridge, MA 02141, USA; 2 Genome Sequencing and Analysis Program, Broad Institute of MIT and Harvard, 320 Charles Street, Cambridge, MA 02141, USA; 3 Genetic Analysis Platform, Broad Institute of MIT and Harvard, 320 Charles Street, Cambridge, MA 02141, USA; 4 Foundation Medicine, One Kendall Square, Suite B6501, Cambridge, MA 02139, USA

## Abstract

Genome targeting methods enable cost-effective capture of specific subsets of the genome for sequencing. We present here an automated, highly scalable method for carrying out the Solution Hybrid Selection capture approach that provides a dramatic increase in scale and throughput of sequence-ready libraries produced. Significant process improvements and a series of in-process quality control checkpoints are also added. These process improvements can also be used in a manual version of the protocol.

## Background

The cost of DNA sequencing continues to fall, driven by ongoing innovation in sequencing technology [1-4]. As a result, it has recently become feasible to sequence non-trivial numbers of whole human genomes [3,5-10]. Many more such projects are planned and commercial genome sequencing services are now becoming available [11,12]. At the same time, there is growing interest in sequencing specific portions of genomes, and several affordable methods for sample preparation of targeted regions have been recently published [13-17]. Key applications for targeted approaches include sequencing of exons or sets of protein-coding genes implicated in specific diseases [18-21], whole human exome sequencing (for example, in cancer or disease cohorts) [22-24] (reviewed in [25]), and resequencing of specific regions as a follow-up to genome-wide association studies [26]. The economics of whole exome sequencing have made targeted enrichment approaches an attractive option for discovery of rare mutations in a variety of diseases as the price tag is substantially lower than for sequencing an entire human genome. For example, using list prices and including the targeted capture step, the all-in cost of sequencing a whole exome (roughly 30 Mb), is 13-fold less than for the whole genome (Table S1a in Additional file 1). This translates directly into a budget that can include more than ten times as many samples, greatly increasing the statistical power of the data to be generated. The effect is even greater for smaller sequencing targets, which further scale down the required sequencing, although costs of targeting scale down more slowly. Ultimately, as long as the expense of the required sample preparation does not dominate, targeting will continue to be a cost-effective approach. To date, however, no targeting method has been described that can handle the many thousands of samples that are becoming available. To fill this need, we set out to develop such a method.

**Figure 1:**
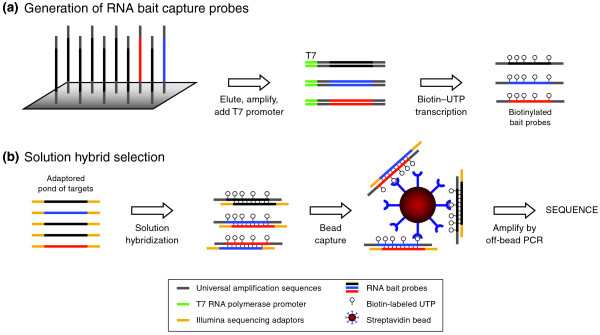
**Overview of the hybrid selection method**. Two specific sequencing targets and their respective capture baits are indicated in blue and red. **(a) **Generation of RNA bait capture probes. 150mer oligos are synthesized on array in batches of 55,000 and cleaved off. They are made double stranded by PCR amplification and tailed with a T7 RNA polymerase promoter, and RNA capture baits are made by transcription in the presence of biotinylated UTP. **(b) **Solution hybrid selection. RNA baits (from the top line) are mixed with a size selected pond library of fragments modified with sequencing adaptors. Hybridized fragments are then captured to streptavidin beads and eluted by the with-bead protocol for sequencing. See text for details.

Solution hybrid selection (SHS), developed by Gnirke *et al. *[14], was created as a tool to cheaply and effectively target multiple regions in the genome in a way that is compatible with next generation sequencing technologies (Figure 1). The published protocol performs well in terms of efficiency of enrichment (selectivity), reproducibility, evenness of coverage, and sensitivity to detect single-base changes [14]. Using this method, a single technician can process six samples simultaneously from genomic DNA to sequence-ready library in approximately one week. This process was designed purely as a series of liquid handling steps and incubations, with the specific intention of making it amenable to scale up and automation. Given the demonstrated success of this and other methods, demand for targeted sequencing has increased sharply. To accommodate the increased demand, keep costs down, and limit the requirements for human labor, we have adapted SHS to an automated high-throughput process. This SHS method includes improvements designed to increase the efficiency of the target selection process through optimization of reactions and automation of the library and capture procedures using liquid handling robots. Several aspects of this method, in particular the 'with-bead' sample preparation method, are amenable to sample preparation steps for a range of next generation sequencing applications, including alternative in-solution and solid-phase capture strategies.

To support high-throughput SHS for targeted sequencing, we set out to devise a laboratory process that would handle very large numbers of samples in parallel for targeting and preparation of sequence-ready libraries at a low cost per sample. This process was designed to carry out whole exome targeting but also yields good results in targeting subsets of genes or regions for resequencing. Results described here come from whole exome targeting using the Agilent SureSelect Human All Exon v2 kit, which is a commercially available implementation of the optimized capture reagent we have described previously [14].

A number of challenges were overcome in developing a robust, automated, and highly scalable process for selection of exomes and other targets. Beyond the need for processing large numbers of samples, modifications of the protocol were made to achieve or maintain the following: elimination of manual, agarose gel-based size selection, which has now been replaced by fully automated, bead-based steps; high selectivity, with a high number of sequenced bases on or near the target region of interest; evenness of sequence coverage among captured targets, avoiding highly overrepresented targets and dropouts; high library complexity, or low molecular duplication, so that libraries contain large numbers of unique genome fragments; reproducibility, so that performance of the process is highly predictable; low cost of the targeting process relative to sequencing; detailed process tracking to reduce errors and provide sample history; quality control checkpoints built into the process to identify poor performers prior to sequencing; and limited human labor.

We present here a scalable, automated SHS method that operates at a throughput far higher than achieved by other methods. The process can also be carried out by hand using a multichannel pipetter. This method has not only been scaled but also optimized to improve selectivity and evenness of target coverage and to minimize artifactual duplication to consistently deliver greater than 94% of the alignable exome (Additional file 2). The automated protocol has a capacity to process over 1,200 SHS samples in less than a week with four technicians (one technician can generate 1,200 pond libraries per week, and three technicians can each generate 384 SHS captures per week). For ease of explanation, we employ a fishing-based terminology in SHS, where the biotinylated RNA capture reagent is referred to as the 'bait', the genomic DNA library from which targets are captured as the 'pond' in which we are 'fishing', and the DNA targets from the pond that are captured by the bait are referred to as the 'catch'.

## Results

### Building a high-throughput solution hybrid selection process

SHS is a method used to selectively enrich for regions of interest within the human genome [14] (Figure 1). Briefly, a library (or 'pond') of adapter-ligated fragments of randomly sheared DNA is hybridized to biotinylated RNA (or 'baits') that are complementary to the target sequences. Hybridized molecules (the 'catch') are then captured using streptavidin-coated beads. Once the captured DNA fragments are PCR amplified off the capture reagent, they are available to be sequenced using next generation sequencing technologies. The standard SHS protocol was redesigned from a manual, bench scale process to an automated process, in much the same way as our recent work to scale library construction for 454 sequencing [27], and is capable of far greater throughput than demonstrated for other methods (Additional file 2). A series of process innovations were required to facilitate reimplementation of this process at large scale. In particular, all manual pipetting steps were converted to automation-amenable liquid handling steps, and these liquid handling steps were extensively optimized to maximize yield efficiency. As part of this, the electrophoretic size selection step has been replaced by fully automated bead-based sizing. Other optimizations are described below. Table 1 shows a comparison of the original published method and the new protocol with a description of each step and the improvements in the new method. Table 2 describes a set of key sequencing metrics by which we measure SHS process performance.

**Table 1:** Comparison of standard versus improved solution hybrid selection methods

	Manual standard SHS protocol		Automated improved SHS protocol	
		
Process step	Standard method	Drawbacks	Improved method	Advantages
Shearing of genomic DNA	Covaris S2	Single sample	Optimized Covaris E210	Multi-sample, improved yield, tight size range
Enzymatic cleanups	Individual spin columns	Low throughput, 50 to 60% recovery, manual	'With-bead' SPRI	High throughput, 80 to 90% recovery, automated
Solution hybrid selection capture	Manual, column- based	Labor intensive (6 samples/FTE/week)	Fully automated	Walkaway, high throughput (1,200 samples/4FTE/week)
Final PCR enrichment	Denature, followed by PCR	Sample loss through transfers	Direct 'off-bead' PCR	Improved final yield
In process quality control checkpoints	Agilent Bioanalyzer	Limited visibility until sequence results	Many	In process results: key predictors of sample, library and sequencing quality

FTE, full time employee; SHS, solution hybrid selection; SPRI, solid phase reversible immobilization.

**Table 2:** Automated solution hybrid selection performance

Performance factor	3 μg input average (*n *= 1,117 exomes)
Median target coverage	131.0×
Percentage bases > 2×	96.0%
Percentage bases > 10×	91.9%
Percentage bases > 20×	87.6%
Percentage selected bases (on target)	83.7%
Percentage duplicated reads	4.4%
Fold 80 penalty^a^	3.17
Estimated library size of captured fragments	278 million

See Additional file 12 for metric definitions. ^a^Fold 80 penalty is a measure of the non-uniformity of sequence coverage, defined as the amount of additional coverage (in fold coverage of the genome) required so that 80% of the target bases will be covered at the current mean coverage (see Additional file 12 for details).

The automated SHS process is implemented on the Bravo liquid handling workstation (Agilent Automation Solutions), a commercially available small-footprint, liquid handling platform, but can be implemented on many commercially available liquid handlers. The process can also be carried out manually using a multichannel pipette. An overview map of the process can be found in Additional file 3 and the manual protocol version can be found in Additional file 4.

### Optimization of acoustic shearing

The process begins with fragmentation of genomic DNA using the Covaris E210 adaptive focused acoustics instrument. Maximizing the yield of DNA fragments in the desired size range is a key step in minimizing overall sample loss. The Covaris E210 instrument focuses acoustic energy into a small, localized zone to create cavitation, thereby producing breaks in double-stranded DNA. A number of variables control mean fragment length and distribution, including duty cycle, cycles per burst, and time. The Covaris adaptive focused acoustics system has several advantages over other methods such as nebulization or hydrodynamic force. First, DNA is sheared in a small closed environment and is not handled in large volume vessels or in tubing, greatly reducing sample loss. Second, the closed, independent vessels greatly reduce sample cross-contamination. Third, the Covaris machine can operate automatically on up to 96 samples per run, eliminating significant sample handling labor and eliminating shearing as a process bottleneck. Fourth, improvements to the shearing protocol in combination with removal of small fragments in subsequent bead-based clean up steps (see below) eliminates the need to size select and extract samples from agarose gels, a critical bottleneck in the overall process.

Shearing performance was extensively optimized for increased sample yield, narrower insert size distribution, and robust and reproducible handling of large numbers of samples in parallel. Optimizations focused on the following factors: shearing volume, tube type, elimination of tube breakage, shearing pulse time, water degassing, and positioning of tubes in the water bath (see Materials and methods for details). In order to accommodate automated handling of the samples, volumes were reduced from 100 μl to 50 μl without any effect on shearing profiles or sample loss (Additional file 5). Importantly, proper fit of the shearing rack (Covaris, catalogue number 500111) into custom adapters (see Additional file 6 for CAD drawing) prevents movement, allowing transfers to occur via automated liquid handling. In addition, specific tubes available from Covaris (Covaris, catalogue number 500114) virtually eliminated the problem of tube breakage. Only a single sample in the most recent 5,000 processed suffered a broken tube. Through a systematically designed and controlled set of experiments, optimal pulse time parameters were chosen to provide a mean fragment length of 150 bp with a range of 75 to 300 bp (Materials and methods). Additional file 5 shows the contrast between unoptimized and optimized size profiles of sheared DNA. In addition to regular maintenance, careful degassing of the water bath and proper water levels are critical for reproducible results. In a nondegassed water bath dissolved oxygen reduces cavitation and disperses energy, reducing shearing efficiency.

### Modified bead-based cleanups enable scale-up to 96 wells

**Figure 2:**
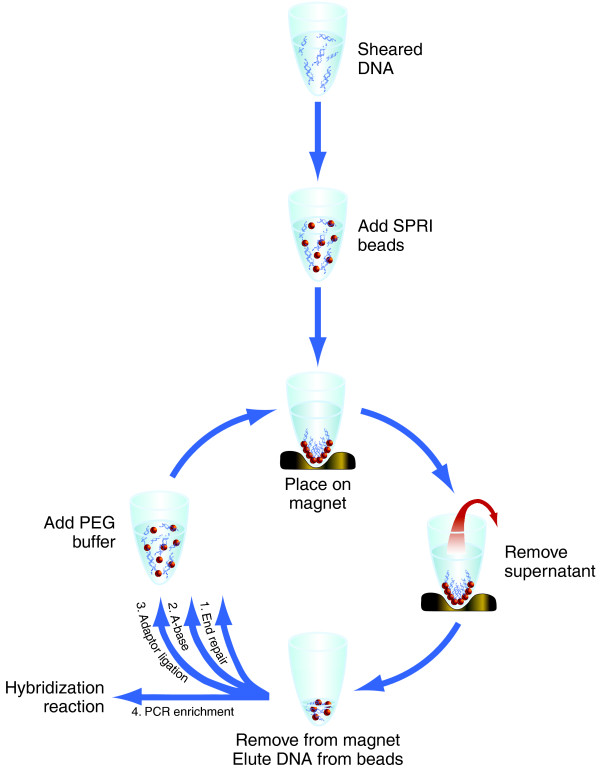
**With-bead SPRI method for pond library construction**. SPRI magnetic beads are added to the sheared DNA sample. DNA is selectively bound to SPRI beads, which are immobilized when the sample plate is placed on a magnet, leaving other molecules in the liquid phase. The liquid phase is removed and discarded. The sample plate is then removed from the magnet and DNA is eluted from the beads. Library construction master mixes are then added to eluant/bead solution. The DNA and SPRI beads then pass through three cycles of reaction, binding to beads (in the presence of polyethylene glycol (PEG)/NaCl solution) and cleanup/washing. The cycles carry out end repair, A-base addition and adaptor ligation, respectively. A final elution is then followed by PCR amplification.

A key requirement in scaling SHS was to implement processing of samples in a standard 96-well microtiter plate. This was facilitated by development of a novel modification to solid-phase reversible immobilization (SPRI) magnetic bead reaction cleanup methodology [27,28] we have termed 'with-bead' SPRI (Figure 2), which is highly scalable due to its amenability to liquid handling automation. Implementation of with-bead SPRI in SHS offers significant advantages. First, it replaces single tube spin-column-based cleanups with liquid handling-compatible magnetic bead-based cleanups; second, it enables selection of molecular weight ranges, eliminating the need for agarose gel-based sizing; third, it simplifies the process by allowing elimination or combining of several steps, which results in a higher overall DNA yield.

**Figure 3:**
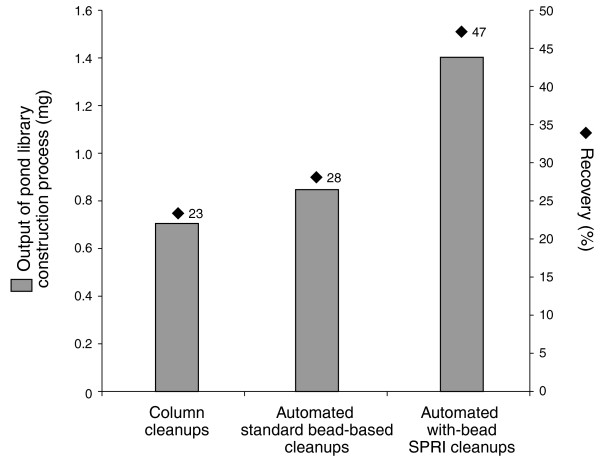
**Yield output from pond library construction methods**. Data are shown left to right, for pond libraries constructed with three methods: the widely used standard column-based cleanups [14], an automated implementation of standard bead cleanups and our implementation of with-bead SPRI cleanups. Each library was constructed with 3 μg input of NA12878 genomic DNA, in triplicate. Bars: total DNA output from pond library construction before PCR amplification. Blue diamonds: percentage recovery of input DNA for duplicates of 3 μg of the same input DNA. With-bead-based cleanups increased the amount of DNA retained throughout library construction compared to the standard column or SPRI cleanup methods.

The innovation of the with-bead SPRI method is as follows. Rather than employing a series of discrete cleanup steps in the library construction process, the cleanups are effectively integrated. The SPRI beads are added to the sample after the shearing step, and remain in the reaction vessel throughout the sample preparation protocol. By allowing each cleanup step to employ the same beads, the with-bead method greatly reduces the number of liquid transfer steps required. The 'cleaned up' DNA is then eluted at the conclusion of the process. This methodology increases the overall DNA yield (Figure 3), primarily because it allowed us to eliminate six of the ten sample transfer steps, avoiding the loss of DNA sticking to the sides of the vessel or loss of volume in pipetting. Briefly, following each process step, DNA is selectively bound to the iron beads, already present, through the addition of a 20% polyethylene glycol (PEG), 2.5 M NaCl buffer. The mixture is placed on a magnet, which pulls the beads and bound DNA to the sides of the well so that the reagents, washes and/or unwanted fragments can be removed with the supernatant. Molecular weight exclusion, which is essentially a size selection, of unwanted lower molecular weight DNA fragments can be controlled through the volume of the PEG NaCl buffer that is added to the reaction, changing the final concentration of PEG in the resulting mixture and altering the size range of fragments bound to the beads [27,28]. DNA fragments that have been cleaned or size selected are eluted from the beads, ready for the next step; however, the eluate is not transferred into a new reaction vessel. Rather, the reagents for the next step are added directly to the reaction vessel containing samples and beads. The presence of beads does not interfere with any of the steps in the process (Table 3). This with-bead protocol has greatly increased the number of unique fragments entering the pond PCR step, increasing the complexity of libraries made by roughly 12-fold (Table 3).

**Table 3:** Performance comparison of manual versus automated solution hybrid selection

Factor	Column based	Automated (with-bead SPRI)	Automated (with-bead SPRI) low input
Input DNA	3 μg	3 μg	0.1 μg
Samples/FTE/week	6-12	384	384
Number of sample transfer steps	10	4	4
Output DNA prior to PCR	720 ng	1,330 ng	Below limit of detection
Number of pond PCR cycles	12-16	6	6
Percentage duplicated reads	19.8	2.2	10
Percentage selected bases	84.7	88.6	83.76
Estimated library size	43 million	516 million	223 million

FTE, Full time employee; SHS, solution hybrid selection; SPRI, solid-phase reversible immobilization.

This increase in yield with the with-bead SPRI protocol has the added benefits of reducing both the input DNA requirement to the process and the number of PCR cycles required. Efficient with-bead targeted captures can be achieved with pond libraries made with as little as 100 ng of input DNA and six to eight cycles of PCR, a major improvement over the commercialized SHS method, which requires 3 μg of starting genomic DNA and 14 cycles (Table 3). We note here that PCR cannot be completely eliminated because the efficiency of adaptor ligation varies between samples, probably because of variation in input DNA quality. PCR cycle number was optimized to maximize the number of unique fragments in the library while minimizing the duplication rate (Additional file 7). This resulted in a modest number of cycles that enriches fragments containing an adapter at each end but not fragments with either no adapters or an adapter at one end only. These incomplete constructs compete with two-adapter fragments in the hybridization reaction but cannot be sequenced.

### Pre-mixed reagents for automated library construction

Currently available commercial library reagent kits are packaged for bench-level processing of eight to ten samples. In order to accommodate the increase in scale and automated processing of samples, large-scale reagent kits were developed and optimized for the high-throughput SHS pond construction process. All buffers and non-enzyme components are premixed and aliquoted at volumes appropriate for 96 samples, including necessary dead volume. Prior to use, the premixed reagents only need to be thawed and placed on the deck where enzymes are added immediately before dispense into reaction plates. To accomplish this, we developed a custom reservoir in combination with optimized aspiration and dispense protocols. The custom reservoir is designed to limit dead volume, thereby minimizing the reagent volume required, thus reducing reagent waste. Details, including the dimensions of the reservoir, can be found in Additional file 8.

### Automation of capture protocol to process 96 samples simultaneously

The most labor-intensive step in the manual selection process is the 'capture' protocol (Table 1), where hybridized DNA-RNA bait duplexes are separated from unbound fragments. The separation is performed using streptavidin beads that bind to the biotin molecules that are covalently linked to the RNA bait. Fragments that are not hybridized to the biotinylated RNA baits are removed through a series of washes.

Wash conditions were redesigned for compatibility with automated liquid handling and optimized for maximal yield (Additional file 9). Since microtiter wells are of much smaller volume than the standard microtubes used in the manual process, the number of wash cycles was increased as the volume of each wash had to be decreased to fit the wells while maintaining the proper level of stringency. Wash buffers are precisely controlled for temperature by storing the buffer-containing vessels in 65°C temperature-calibrated heating blocks (V&P scientific, VP-741BW MICA) integrated onto the deck of the liquid handler robot. This automation provides a hands-off capture protocol capable of consistently setting up capture reactions for 96 samples in 4 hours; in comparison, the manual (and somewhat variable) process handled 6 samples in 4 hours. Additionally, the automated process delivers output of a more consistent quality, and eliminates manual tracking and pipetting errors (Additional file 10).

### Off-bead PCR to increase yield of captured product

In the manual protocol [14], the elution of desired DNA fragments from the RNA bait-streptadavidin bead complex is accomplished by denaturation using 0.1 N sodium hydroxide followed by a cleanup step prior to PCR amplification. This series of steps requires large volumes and is therefore difficult to scale in a microtiter plate format. In addition, variability at this step can result in loss of captured DNA. We have replaced elution through denaturation by amplifying the captured sequences directly by PCR, by a process we term 'off-bead' PCR, as the target is PCR amplified off the bead directly in the capture plate. This allows scaling in a microtiter plate format, simplifies the process by removing a pipetting step, eliminates process variability and improves the yield of captured product roughly three-fold (Additional file 11). Briefly, PCR enzyme, PCR primers, and dNTPs are added directly to the bead-bait-DNA complex, and the mix is amplified via thermalcycling (see Materials and methods for details). Bait RNAs, which lack Illumina adapter sequences, and pond fragments with fewer than two adapters are not amplified. The amplified fragments are then separated from the beads through a modified SPRI bead cleanup (Materials and methods). This off-bead PCR protocol, in combination with improvements described above, significantly improves yield at this step in the process (Table 3). This simple, automation-friendly, cost-effective protocol can be used to process up to 1,200 samples per week in batches of 96 (Table 2).

### Development and automation of in-process quality control checkpoints

**Figure 4:**
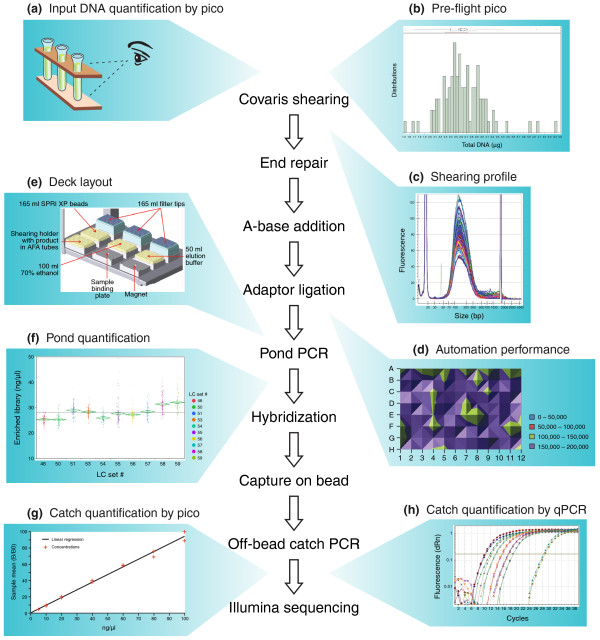
**Quality control checkpoints**. **(a-h) **Eight different quality control checkpoints for the scaled SHS process are schematized. Quality is assayed at key steps to quickly identify failed samples and also to provide ability to troubleshoot process failures. See text for details. AFA, adaptive focused acoustics.

As the process increases in scale, readouts of sample quality and process success become increasingly important as indicators of the likelihood of producing high quality sequencing results. To this end we have implemented a series of in-line quality control checkpoints. This enables granular reporting of metrics during the SHS process and, importantly, allows poorly performing samples to be quickly identified and removed, avoiding the associated costs of downstream processing and sequencing (Figure 4). Central to this is the development of critical quality control assays, both in terms of their sensitivity to the samples at the point at which they are assayed, as well as their utility as a predictor of sequencing quality. The eight key quality control checkpoints that add immediate value to the process are outlined below (see Materials and methods for details on each).

#### Volume check

Volumes are checked for every sample by visual inspection to ensure predictable performance in shearing (Figure 4a). If volumes are outside of specification (50 μl ± 20%), samples are either concentrated or diluted to reach the appropriate range. Low volumes cause inaccurate automated transfer of sample into shearing vessels.

#### Sample concentration check by PicoGreen

Concentrations for all samples are measured via an automated PicoGreen assay (see Materials and methods) and are specified to be within 2.0 to 60 ng/μl (Figure 4b). Samples above this range are normalized and re-aliquoted to appropriate volumes since excess input DNA can actually inhibit the enzymatic pond reactions (data not shown). Samples above the 2.0 ng/μl threshold are considered to pass. Those below this range can be run on risk.

#### Size quality control of sheared DNA

Sheared samples are assayed on an automated microfluidic electrophoresis instrument, the Caliper GX system, using the 1K DNA Chip to evaluate the size distribution produced by the Covaris instrument (Figure 4c). Fragment sizes should be between 75 and 300 bp with the distribution centered on 150 bp. Samples that shear above this range can decrease the specificity and efficiency of the selections. Samples sheared to less than a mean of 110 bp will be suffer losses during the various with-bead cleanups, greatly reducing the complexity of the library before selection.

#### Performance quality control of automation

The Bravo automated liquid handling platform is assayed daily for dispense accuracy and precision using a quantitative fluorescent dye assay (Figure 4d). Standard liquid handling sequences are run using sulforhodamine dye, and relative fluorescent units of the dispensed dye are assayed on a Perkin Elmer Victor3 plate reader. Coefficients of variation (%CV) are calculated between wells and must be within three standard deviations of the mean. If the robot is out of specification, maintenance is performed on the system followed by repeat of the quality control until the coefficients of variance are back within acceptable ranges.

#### Confirmation of deck configuration

To confirm proper set up of the Bravo platform before each step in the protocol, the software requests the operator to confirm the proper deck layout by comparing the deck positions to a picture shown on screen (Figure 4e). This prevents users from starting programs without the proper materials in place or from running the wrong combination of program and deck configuration.

#### Quantification of pond libraries and catch libraries

Prior to selection, pond libraries are assayed for concentration by an automated PicoGreen assay (Materials and methods) and are specified to be within a range of 25 to 60 ng/μl in a volume of 40 μl (Figure 4f). Samples at concentrations greater than 25 ng/μl are normalized to 25 ng/μl prior to hybridization. Samples below this 25 ng/μl generally produce sequence data with high amounts of duplication. After capture, samples are again assayed in a similar fashion (Figure 4g). All catches with concentrations greater than 5 ng/μl are passed on to the next step in the process. Catches with concentrations less than 5 ng/μl are considered failures and can be sent for re-selection.

#### Quantitative PCR quantification of catch

Final evaluation of the catch material employs an automated quantitative PCR assay developed in conjunction with Kapa Biosystems (KAPA Library Quantification Kit, catalogue number KK4832) designed to accurately quantify the fragments containing two Illumina adapters (Figure 4h). This step is critical for determining the correct concentration of the library to be loaded for sequencing on the Illumina platform, to maximize cluster densities and sequencing quality. Samples at concentrations greater than 2 nM have been found to produce sequencing data with sufficient complexity.

In addition to the in-line assays, each 96-well plate of samples contains control DNAs (two positives and one negative) that are used for quality assessment (see Materials and methods). The control checkpoints established throughout the process provide early warning of issues with performance of each step and overall quality. In addition to these in-process lab assays, we have developed a number of key sequencing metrics that allow us to gauge the success of each selection (Table 3) as well as the performance of the process over time (Additional file 10) in support of continuous process improvement and optimization (see Additional file 12 for further definition of sequencing metrics).

### Sample tracking and integrity

Any process that handles large numbers of samples must have a supporting sample tracking system that preserves sample identification and manages association of critical process data necessary for analysis. As part of the scaled SHS process, we developed and implemented a comprehensive tracking system that associates sample information with a unique barcode on each sample tube and microtiter plate. Every step takes place in barcoded plasticware, and each step where samples are moved is associated with a barcode scan that is reported to the database so that data trails across all sample handling events are complete. Microtiter plates are labeled with unique code 128 barcodes, and individual sample tubes are labeled with two-dimensional data matrix barcodes. This system provides flexibility to associate unique information with samples, providing granular tracking and the ability to track sample progress at the plate level. Samples can thus enter the process from static 96-well plates or from individually barcoded two-dimensional tubes in a 96-well rack layout. Two-dimensional barcodes are read by a flatbed data-matrix barcode scanner (BioRead-A6, Ziath Ltd, part number 2002Z), integrated into both our custom laboratory information management system and the Bravo 96-channel liquid handling robot.

In addition to comprehensive tracking of sample handling, for human DNA samples we have developed an additional layer of control to ensure that the DNA sequence data ultimately delivered matches the exact input DNA sample. Briefly, 24 baits that specifically capture well-characterized human polymorphic sites are supplemented into the Agilent SureSelect Human Exon v2 bait reagent before SHS. SNP calls derived from resulting exome sequencing data are then compared to previously generated genotype data for absolute validation of biological sample identity. The baits capture 22 SNPs on the autosomes, one SNP on chromosome X and an indel on chromosome Y that acts as a gender assay (one allele being fixed on X and the other fixed on Y), and together are highly diagnostic of identity. The sequences of the 24 baits are available in Additional file 13.

After sequencing and mapping of data to the genome, the genotypes of these 24 loci are determined using a simple quality-aware Bayesian genotyping algorithm similar to published tools [29,30] and compared to those previously ascertained using a genotyping technology such as the Sequenom HME platform or the Affymetrix SNP 6.0 platform. These results are used to confidently confirm or reject sample identity, ensuring that the likelihood of having incorrectly confirmed sample identity is on the order 1/100,000 at worst and several orders of magnitude less likely at best. Human samples for which identity has been rejected are checked against all human samples in our genotype database, and in virtually all cases the mistaken identity can be clarified.

## Discussion

**Figure 5:**
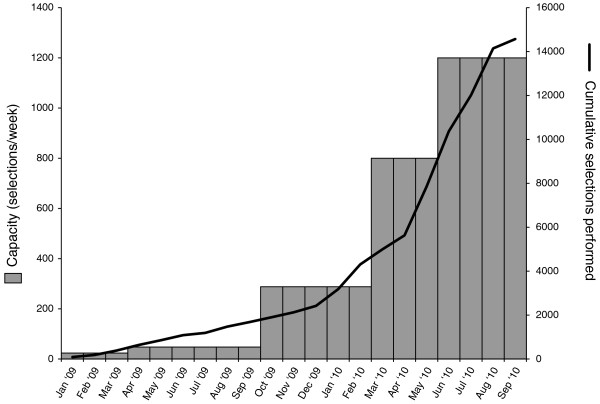
**Increasing capacity over time and cumulative output**. Bars show capacity for selections per week of protocols by date. Line shows cumulative hybrid selection captures performed.

Targeted sequencing is a powerful approach. By enabling sequencing of only the desired regions of a genome it provides a significant reduction in cost per sample over whole genome shotgun sequencing. For example, capture and sequencing of a complete human exome can be done at a cost of roughly 10- to 20-fold less per sample than whole genome shotgun sequencing. Early success of targeted sequencing methods [13,18-23,26] has created a rapidly growing demand for targeted sequencing in areas such as cancer, human genetic disease, and validation of genome-wide association studies. In such projects the number of samples required to get meaningful statistical power, often hundreds or thousands, makes whole genome sequencing prohibitively costly. To meet this demand, we have adapted the SHS method of Gnirke *et al. *[14] so that it can be performed at high scale on an automated platform allowing a single technician to perform 96 simultaneous capture events in standard microtiter plate format. The method maintains the high selectivity and high library complexity of the original manual process, delivering selected sequence reads with a high on-target rate of > 83%, and a median rate of duplicated reads of approximately 4%, similar to that of whole genome shotgun sequencing (Table 2). Figure 5 shows the increase in capacity of the SHS process over time, to a current level of 1,200 samples per week, and also shows output for the automated process, with a cumulative total of over 14,000 samples processed.

SHS is particularly amenable to scaling and automation because the entire protocol is a series of liquid handling events. We have successfully implemented it as a highly scaled process on a standard laboratory liquid handling platform. Automated protocols can be found in Additional files 14 and 15. As part of automation and scaling of SHS, we have introduced a series of innovations and optimizations to the original manual process, including: optimization of shearing, gel-free size selection, 'with-bead' sample preparation, 'off-bead' PCR and a series of in-process quality control checkpoints. The shearing step was optimized to maximize yield of fragments in the desired size range, to be compatible with the subsequent gel-free size selection step and configured to be carried out in a 96-well format. For sample cleanup and removal of unwanted small fragments, we devised a novel 'with-bead' method, in which the magnetic beads used for isolating the DNA remain in the well with the sample through a series of steps. This is a key innovation, as it eliminates a large number of liquid handling steps, greatly reducing sample loss.

The improvements described here are not limited in application to SHS. Each can be applied to a wide variety of sample preparation processes for next generation sequencing, and to any of the sequencing technology platforms available. This 'with-bead' protocol in particular is a widely applicable approach as it can be used to increase scale and reproducibility, and to reduce input DNA requirements. In particular we are using it for production library construction for both Illumina and 454 sequencing, and for construction of libraries for ChIPseq. It can also be used for other capture methods such as the NimbleGen liquid phase (SeqCap EZ) method.

PCR enrichment and hybridization capture steps were optimized to greatly increase yield and to minimize amount of off target and duplicated sequences delivered. A series of in-process quality control checkpoints has been added to permit detailed monitoring of the process and support continued optimization. These granular quality control checkpoints allow easy identification of problems, such as bad reagent lots, robot performance issues or poor quality samples, before the expensive sequencing step takes place. Finally, the process includes comprehensive sample tracking via end-to-end sample barcoding, virtually eliminating sample handling and tracking errors. Importantly, the scalability of the SHS method means that we can comfortably produce libraries at a higher rate than they can typically be sequenced, preventing sample preparation from becoming a bottleneck.

The scaled SHS process, as currently implemented, utilizes a 96-well format in the hands of a single trained laboratory technician, but can easily be scaled to larger numbers with the addition of plate stacker hardware. For example, using this configuration our group currently has the capacity to carry out roughly 1,200 sample preparations per week with a team of four technicians. For modest throughput, the extensive technical improvements of the optimized SHS process can also be carried out by hand with a multichannel pipette. Though not approaching the scale of the automated process, this still represents a significant improvement in ease of use, scale and efficiency over the standard process.

Application of targeted sequencing is becoming widespread, and has been successfully demonstrated as described in recent publications [13,18-23,26]. Following close on the heels of these early successes, large numbers of studies are now ready to apply targeted sequencing, particularly in the areas of cancer and human genetic disease. For efficient and cost-effective targeted sequencing of large numbers of samples, an automated, large scale and fully tracked targeted sequencing process is essential. We have described here the first such process, which makes this approach straightforward for very large numbers of samples. Partly as a result of this, targeted sequencing is poised to have a transforming effect on medical and cancer genomics in the near future.

## Materials and methods

### Shearing of genomic DNA

In sets of 96, 50 μl aliquots of purified genomic DNA were transferred using the Bravo liquid handling platform (Agilent Automation, Santa Clara CA, USA, catalogue number 5400A) from 0.5 ml two-dimensional barcoded tubes (ThermoFisher Matrix, Hudson NH, USA, catalogue number 3744) into glass microtubes (Covaris, Inc., Woburn MA, USA, catalogue number 500114) held in a 96-well rack (Covaris, Inc., catalogue number 500111). A specially designed adapter to hold the 96-well rack was used (CAD design available in Additional file 6) to prevent disposable tips from lifting the rack off the plate pad of the Bravo platform, which makes them susceptible to breakage. Samples were sheared for 165 s at Duty cycle = 20%, Intensity = 5, Cycles per burst (CPB) = 200, Z-axis = 0 mm). The water bath level should come halfway up the tube. Complete degassing of the water (coupling fluid) prior to shearing is critical. The degas pump should be turned on 30 minutes prior to shearing.

### Pond library construction

All liquid handling steps were carried out on the Bravo liquid handling platform using VWorks Automation Control Software (Agilent Automation, Santa Clara, CA, USA). Enzyme mastermix dispenses were performed using the Bravo configured with the 96ST pipetting head using 70-μl disposable tips (Agilent Technologies, catalogue number 19133-102), and a custom adapter (see Additional file 8 for CAD designs) to hold disposable reagent reservoirs (Labcyte, Inc. Sunnyvale, CA, USA, catalogue number ALL031-01). All SPRI cleanup steps were performed using the Bravo configured with the 96LT pipetting head and 180 μl disposable tips (Agilent Technologies, catalogue number 08585-002).

Sheared fragments were cleaned up using SPRI Ampure cleanup by adding 150 μl of SPRI AMPure XP (Beckman Coulter Genomics, Danvers, MA, USA, catalogue number A63881) beads to the shearing vessel. After mixing, the bead-DNA mixture was transferred to a standard 96-well PCR plate (Eppendorf, Hamburg Germany, catalogue number 47744-116) for the remainder of the library construction process. A general SPRI cleanup involves addition of SPRI beads suspended in buffer containing 20% PEG and 2.5 M NaCl to DNA reaction products. After thorough tip mixing and a 2-minute incubation at ambient temperature, the plate was transferred to a magnet plate (Life Technologies, Carlsbad, CA, USA, catalogue number DYNAL MPC-96S), incubated for 4 minutes at ambient temperature, and the supernatant was removed. Beads were washed with 100 μl 70% ethanol, the plate was moved off the magnet, and the beads were dried for 6 minutes at room temperature. Desired DNA fragments were eluted off the beads through the addition of 40 μl 10 mM Tris-HCl pH 8.0. Additional details, including specific reagent volumes, are included in Additional file 14.

Reagent kits are prepared in advance for enzymatic steps including end repair (New England Biolabs Ipswich, MA, USA, catalogue number M0201B-96, M0203B-96), A-base addition (New England Biolabs, catalogue number M0212B-96), and ligation reactions (New England Biolabs, catalogue number M2200B-96). See supplementary material for detailed protocols for the manual and automated implementations of the process (Additional files 4, 14).

### Optimization of pond PCR to enrich for fragments with proper adapters

Optimized PCR enrichment conditions were performed by adding the following to 40 μl of eluted DNA from the adapter ligation reaction: 4 μl of Illumina F&R PE Enrichment Primers (Illumina, Inc., San Diego, CA, USA, catalogue number 1002290), 1 μl 100-mM dNTP mix (25 mM each; Agilent Technologies 200415), 6 μl 10× buffer (0.1 M KCl, 0.01 M MgSO_4_.7H_2_O, 0.01 M bovine serum albumin, 0.01 M (NH_4_)_2_SO_4_, 0.2% Tris-HCl, 0.001% Triton X-100), 2 μl Pfu Ultra II Fusion HS DNA Polymerase (Agilent Technologies, catalogue number 600852) and 7 μl nuclease free water (VWR, Radnor, PA, USA, catalogue number PAP1193). Reactions were incubated on Eppendorf Mastercycler Pro thermalcyclers (Eppendorf, catalogue number 6321 000.515) for 120 s at 95°C, and cycled six times for 30 s at 95°C, 30 s at 65°C and 60 s at 72°C.

### Hybridization and capture of pond fragments to RNA baits

Twenty microliters of pond libraries diluted to 25 ng/μl were hybridized using whole exome baits (Agilent SureSelect Human All Exon Kit v2). The reaction was carried out according to manufacturer's specifications for the SureSelect Target Enrichment System Sequencing Platform Library Prep v2.0 (Agilent Technologies, catalogue number G3360-90000). Additional fingerprint baits used to check sample identity were prepared according to the published protocol [14] and spiked into the whole exome bait reagent prior to hybridization.

Hybridization buffer, pond libraries with spiked in blocking agents, and bait aliquots were aliquotted to separate 96 well Eppendorf Twintec plates (catalogue number 128.648). This was carried out on the Bravo liquid handling platform outfitted with the 96ST pipetting head using 70-μl disposable tips (Agilent Technologies, catalogue number 19133-102).

Hybridization was carried out by denaturing the plate for 95°C for 5 minutes and then incubating for 72 hours at 65°C on an Eppendorf Mastercycler Pro thermalcycler (Eppendorf, catalogue number 6321 000.515).

M280 Streptavidin Dynabeads (Life Technologies, Carlsbad, CA, USA, catalogue number112-05D) were prepared for use by buffer exchange using a modified, scaled protocol that utilized a magnetic separator (Life Technologies, Dynamag-15, catalogue number 123-01D) designed to hold 15-ml test tubes (VWR, catalogue number 21008-917). See automated protocol in supplementary material for details (Additional file 14).

### Automation of capture protocol

Capture of DNA-RNA complexes was performed using the Bravo configured with the 96LT pipetting head, one low plate pad at position 2, and plate heaters (V&P scientific, San Diego, CA, USA, VP-741BW MICA) at positions 2 and 7. All liquid handling steps used 180-μl disposable tips (Agilent Technologies, catalogue number 08585-002). Reactions were carried out according to manufacturer's specifications in the SureSelect Target Enrichment System Sequencing Platform Library Prep v2.0 (Agilent Technologies, catalogue number G3360-90000). Wash protocols were modified to increase the number of wash iterations while decreasing wash buffer volumes to allow wash steps to take place in microtiter plates. See automated protocol in supplementary material for details (Additional file 14).

### Off-bead catch PCR

DNA fragments were released from the biotinylated RNA baits through off-bead PCR amplification. Reactions were carried out by adding 50 μl PCR Mastermix (41.5 μl Ultrapure water, 2 μl Illumina PE enrichment primers, 0.5 μl 100-mM dNTP mix, 5 μl 10× buffer (0.1 M KCl, 0.01 M MgSO_4_.7H_2_O, 0.01 M bovine serum albumin, 0.01 M (NH_4_)_2_SO_4_, 0.2% Tris-HCl, 0.001% Triton X-100), and 1 μl of Pfu Ultra II Fusion HS DNA Polymerase) to Dynabead M280 Streptavidin beads (Life Technologies, catalogue number 112-05D) and incubated on Eppendorf Mastercycler Pro thermalcycler (Eppendorf, catalogue number 6321 000.515) for 120 s at 95°C, cycled 20× for 30 s at 95°C, 30 s at 65°C and 60 s at 72°C and then incubated for 10 minutes at 72°C. PCR reaction products were again purified using SPRI protocol.

### Quality control checkpoints

All quality control assays involved the automated transfer of sample aliquots to 96-well plates using Bravo Liquid Handling platform outfitted with 96ST pipetting head.

#### DNA quantification by PicoGreen fluorescence

DNA samples were quantified at several points throughout the process using PicoGreen fluorescence using Molecular Probes Quant-IT broad range dsDNA kit (Life Technologies, catalogue number Q33120#). Aliquots (1 μl) were transferred into Costar 96-well fluorescence plates (Corning Corp., Corning, NY, USA, catalogue number 3915) along with manufacturer-supplied DNA standards. Fluorescence was measured using a Victorx3 Plate reader (Perkin Elmer, Waltham, MA, USA, catalogue number 2030-0030) with integrated stacker and barcode reader, compared to the standard curve provided in the Quant-IT kit, and analyzed using Workout software (Perkin Elmer, Waltham, MA, USA).

#### Caliper GX DNA sizing assay

Following fragmentation with the Covaris instrument, 3-μl sample aliquots were diluted with 12 μl of Tris-HCl pH 8.0 for a total volume of 15 μl. Aliquots were analyzed for fragment size distribution relative to supplied marker, which is also diluted 1:5 on the Caliper Labchip GX System and v2 software (Caliper LifeSciences, Hopkinton, MA, USA, catalogue number 122000) using a HT DNA 1K LabChip (Caliper LifeSciences, catalogue number 760517).

#### Quantitative PCR

Quantification of adapter-ligated fragments was performed according to the KAPA Library Quantification Kit (KAPA Biosystems, Cape Town, South Africa, catalogue number KK4832). Samples were analyzed in triplicate along with manufacturer-supplied standards in 384 fluorescence plates (Costar, catalogue number 8281) using an Applied Biosystems Prism 7900HT Fast Real Time QPCR system and supplied SDS software (Life Technologies, catalogue number 4329001).

#### Robot performance quality control by dye handling

Precision performance of the liquid handling robot is maintained by regular quality control. A dummy run is performed daily in which 5 μl of a 0.1-M solution of sulforhodamine dye (Life Technologies, catalogue number S-359) is dispensed into each well of a 96-well plate (Eppendorf Twintec). Accuracy is evaluated by measuring fluorescence on the Perkin Elmer Victor ×3 Plate reader (Perkin Elmer, catalogue number 2030-0030). Coefficients of variation are measured for each plate tested, data are stored for trending analysis, and outlying wells (> 3 standard deviations from the mean) are identified. Corresponding barrels on the pipetting head are visually inspected for wear and replaced when necessary.

### Control samples

Each 96-well plate of samples to be processed contains three samples that serve as process controls. These aid in the characterization of potential fail modes. During the sample preparation process, 3 μg of human DNA (Coriell Institute, Camden, NJ, USA, catalog number NA12878) is added to one well in each plate. This highly sequenced individual serves as a positive control. Similarly, 500 ng of a known high performing SHS pond library is added to one well to serve as a control sample for the hybridization process. Finally, one well contains no DNA and serves as a control for cross-contamination in the process.

## Abbreviations

bp: base-pair; CAD: computer aided design; PEG: polyethylene glycol; SHS: solution hybrid selection; SNP: single nucleotide polymorphism; SPRI: solid-phase reversible immobilization.

## Authors' contributions

SF and AB managed the process development, process implementation, and jointly drafted the manuscript. JA supervised implementation and drafted the Materials and methods section. BM worked on automation scripts, developed with-bead cleanup protocols, and contributed significantly to the manuscript and figures. AA, TD, CF, FJ, JN, and ZZ participated in the process development and implementation of protocols and all contributed significantly to the manuscript. LA managed samples and project-specific deliverables. AB and SS provided analysis support and details for Table S1B in Additional file 1. BB advised and participated in development efforts. KC advised and provided analysis on early development efforts. TF managed the development of the analysis Picard pipeline, fingerprinting process controls, and advised on development and implementation of protocols. RS developed custom adapters and machined parts. JS, JW, BR, JT, and AZ developed LIMs automation and sample tracking. RJ and GY managed bait development and contributed to the manuscript. SG oversaw the project and managed samples entering the pipeline. RN oversaw the project, and advised on development work and implementation. CN oversaw the project and managed the writing process. All authors read and approved the final manuscript.

## Supplementary Material

Additional file 1 **Table S1a and S1b - cost comparison**. **(a) **Cost model comparison of whole genome shotgun to whole exome sequencing. **(b) **Performance metrics of whole genome shotgun compared to whole exome sequencing with a control sample. Click here for file

Additional file 2 **Comparison of targeted capture methods**. Table comparing scaled solution hybrid selection to other approaches. Click here for file

Additional file 3 **Automated SHS process map**. A powerpoint file showing a process map for the solution hybrid selection method. Click here for file

Additional file 4 **Manual SHS protocol**. A word document outlining the manual protocol. Click here for file

Additional file 5 **DNA shearing optimization**. Profiles of sheared genomic DNA from unoptimized (blue) and optimized (red) conditions are shown. The size distribution from optimized conditions has a larger fraction of product DNA in the desired size range of 120 to 150 bases. The sharp peaks at approximately 20 and approximately 1,500 bases represent size standards. Click here for file

Additional file 6 **Shearing rack CAD drawing**. A PDF showing the CAD drawing and dimensions for the shearing rack adapter for the Covaris unit. Click here for file

Additional file 7 **Optimization of pond PCR cycle number**. For each number of PCR cycles tested, red bars (left-hand y-axis) show number of unique molecules per library, in millions; green bars (right-hand y-axis) show percent duplicated sequences. Data were generated in a controlled experiment using high quality human female DNA purchased from Promega (Madison WI, USA, catalogue number G1521). Patient samples typically demonstrate lower performance likely due to lower sample quality. Click here for file

Additional file 8 **Reagent reservoir CAD drawing**. A PDF showing the CAD drawing and dimensions for the low volume custom reservoir used for reagent dispensing. Click here for file

Additional file 9 **Optimization of hybrid selection wash conditions**. Results for three sets of conditions are shown: manual protocol from Gnirke *et al. *[14], with three 500-μl washes; unoptimized automated protocol, with three 150-μl washes; optimized automated protocol, with six 150-μl washes. Shown are percent sequenced bases on target for a controlled bait set. Click here for file

Additional file 10 **Improved process control with transition from manual to automated capture**. Implementation of the automated capture protocol greatly reduced sample to sample variability as measured by the percent of bases on or near the target. Data from 550 samples from the production process are shown. Samples in the gray box (the first 110) were performed manually, and samples on the white background represent the first group run with the automated protocol. Click here for file

Additional file 11 **Comparison of DNA recovery between manual NaOH denaturation and automated 'off-bead' enrichment**. Total yield of DNA in nanograms is shown. Click here for file

Additional file 12 **Sequencing metrics definitions**. A Word document that defines the sequencing metrics used to measure process performance. Click here for file

Additional file 13 **Fingerprint bait sequences**. A Word document listing the sequences of baits used in the fingerprint panel. Click here for file

Additional file 14 **Automated SHS library construction protocol**. A Word document detailing the automated SHS library construction protocol. Click here for file

Additional file 15 **Automated SHS hybridization and capture protocol**. A Word document detailing the automated hybridization and capture protocols. Click here for file
