# Supplementary material for: A scalable, fully automated process for construction of sequence-ready human exome targeted capture libraries
Source: Genome Biol. 2011 Jan 4;12(1):R1. doi: 10.1186/gb-2011-12-1-r1 (PMC3091298; doi:10.1186/gb-2011-12-1-r1)
Supplement: Additional file 1 — Table S1a and S1b - cost comparison. (a) Cost model comparison of whole genome shotgun to whole exome sequencing. (b) Performance metrics of whole genome shotgun compared to whole exome sequencing with a control sample. [file gb-2011-12-1-r1-S1.DOCX]

**Supplementary Table 1a. Cost model comparison of whole exome and whole genome shotgun (WGS) sequencing^1^**

|  | **Whole exome** | **WGS** |
| --- | --- | --- |
| Mean PF sequence coverage^2^ | 131x^2^ | 30x^3^ |
| PF Illumina data required to reach coverage (Gb) | 7 | 120 |
| Average Gb per lane^4^ | 5.875 | 6.25 |
| TotaI lanes required to reach coverage | 2 | 20 |
| Paired read length (bases)^5^ | 76 | 101 |
|  |  |  |
| Cost of sample prep ($)^6^ | 100 | 100 |
| Cost of sequencing ($)^6,7^ | 529 | 9900 |
| Cost of capture reagents ($)^8^ | 450^9^ | N/A |
| Total consumable cost (sum of above 3 rows) ($) | 1029 | 10,000 |
| Machine cost per sample ($)^10^ | 301 | 6413 |
| Total sample prep and sequencing cost per sample ($) | 1380 | 16,413 |
| Cost relative to whole exome shotgun | 1x | 11.9x |

**Supplementary Table 1b. Performance metrics comparison of whole exome and whole genome shotgun (WGS) sequencing of NA12878^11^**

|  | **Whole exome** | **WGS** |
| --- | --- | --- |
| Total sequence (bases) | 5,552,431,507 | 86,594,021,638 |
| Average fold coverage (X) | 168.4 | 27.96 |
| Selected bases (%)^12^ | 86.60 | 100 |
| Bases covered <20x (%) | 92.70 | 85.40 |
| Bases covered <10x (%) | 95.80 | 91 |
| Bases covered <2x (%) | 98.80 | 91.90 |
| Concordance with known SNPs (%)^13^ | 1.4^13^ | 3.4 |
|  |  |  |

1. Data from Illumina HiSeq instrument operation.

2. Based on 1130 recent production samples described in this work.

3. Based on 40 recent human WGS samples in production within the Broad Institute Genome Sequencing Platform.

4. Based on 47 Gb per run, which is the average production output for August 2010.

5. Whole exome sequencing uses 76-base reads rather than 101-base reads because sequence construct inserts are, on average, 150 bases in length.

6. Based on Illumina list price as of September 1, 2010.

7. Includes flowcell and reagents for sequencing and cluster generation.

8. Based on Agilent list price as of September 1, 2010.

9. Cost is $300 at a scale of 10,000 or more, making the ratio of WGS to whole exome 13.3x.

10. Based on list price of the machine as of September 1, 2010, assuming 90% up-time, 365 days/year operation, and 3-year amortization. Run time for 76-base paired reads = 8 days. Run time for 101-base paired reads = 10 days.

11. WGS and whole exome targeted sequence data are from NA12878 DNA (Corriell Institute, cat. #NA12878), prepared from cell line GM12878, derived from a European female in the CEPH collection. NA12878 was chosen for this work because is has a large available data set of SNP calls that can be used as a reference set for comparison.

12. Selected bases is defined for the exome as bases in reads aligning to capture bait sequences plus the flanking 250 bases.

13. Truth set is combination of SNP calls from NCBI [33] 1000 Genomes [10], and dbSNP [34].
