# Supplementary material for: A scalable, fully automated process for construction of sequence-ready human exome targeted capture libraries
Source: Genome Biol. 2011 Jan 4;12(1):R1. doi: 10.1186/gb-2011-12-1-r1 (PMC3091298; doi:10.1186/gb-2011-12-1-r1)
Supplement: Additional file 2 — Comparison of targeted capture methods. Table comparing scaled solution hybrid selection to other approaches. [file gb-2011-12-1-r1-S2.DOCX]

**Supplementary Table 2. Quality performance of scaled hybrid selection is similar to or**

**better than that of other approaches, and at much greater scale**

|  | **This work** | **Ng *et. al [22]*** | **Choi *et. al [13]*** | **Bainbridge *et. al [35]*** | **Summerer *et.al [20]*** | **Nikopoulos  *et al. [18]*** | **Hedges *et al. [24]*** |
| --- | --- | --- | --- | --- | --- | --- | --- |
| Type of capture  method | Solution | Array | Array | Solution | Array | Array | Array |
| Number of samples described | 1117 | 12 | 15 | 4 | 2 | 1 | 8 |
| Sequencing target | Exome | Exome | Exome | Exome | 115 cancer-related genes | FEVR related genes | Exome |
| Sequencing target  size (Mb) | 32 | 2.8 | 34 | >30 | 9.2 | 2.5 | 33 |
| Sequencing technology | Illumina | Illumina | Illumina | Illumina | Illumina | 454FLX | 454FLX |
| Read length (bases) | 76 | 75 | 75 | 75 | 36, 50 | Not reported | 340^3^ |
| Paired reads (Y/N) | Y | N | Y | Y | Y | N | N |
| % on target^1^ | 83.7 | 49 | 49 | 78 | 89.4/80.5 | 90.5 | 77.9 |
| % duplicated reads^2^ | 4.4 | 41 | Not reported | 8.3 | Not reported | Not reported | 2.4^3^ |
| Mean depth of  coverage (per base) | 133 | 51 | 43.6 | 52 | Not reported | 13.2 | 8.82 |
| % of target covered  10-fold (X) | 92 | Not reported | 93 | 90.8 | 84.9 | 56.5 | 50 |
| Average data  per sample (Gb) | 0.7–1.3 | 0.7–1.2 | 0.7–1.1 | 0.7–1.0 | 0.7–1.1 | 0.7–1.2 | 0.7–1.3 |

1. Defined locally for each publication. This generally refers to bases aligning to the capture sequences and those immediately adjacent.

2. Defined locally for each publication. Duplicated reads generally refers to reads with identical start sites above those that would be expected by statistical chance. Level of accuracy of this measure depends on read type, read length, depth of coverage used, and whether read pairing information is accounted for.

3. Calculated from reported numbers by dividing the number of mapped bases by average read length and comparing to the number of unique reads.
