# Supplementary material for: A scalable, fully automated process for construction of sequence-ready human exome targeted capture libraries
Source: Genome Biol. 2011 Jan 4;12(1):R1. doi: 10.1186/gb-2011-12-1-r1 (PMC3091298; doi:10.1186/gb-2011-12-1-r1)
Supplement: Additional file 3 — Automated SHS process map. A powerpoint file showing a process map for the solution hybrid selection method. [file gb-2011-12-1-r1-S3.PPTX]

## Slide 1
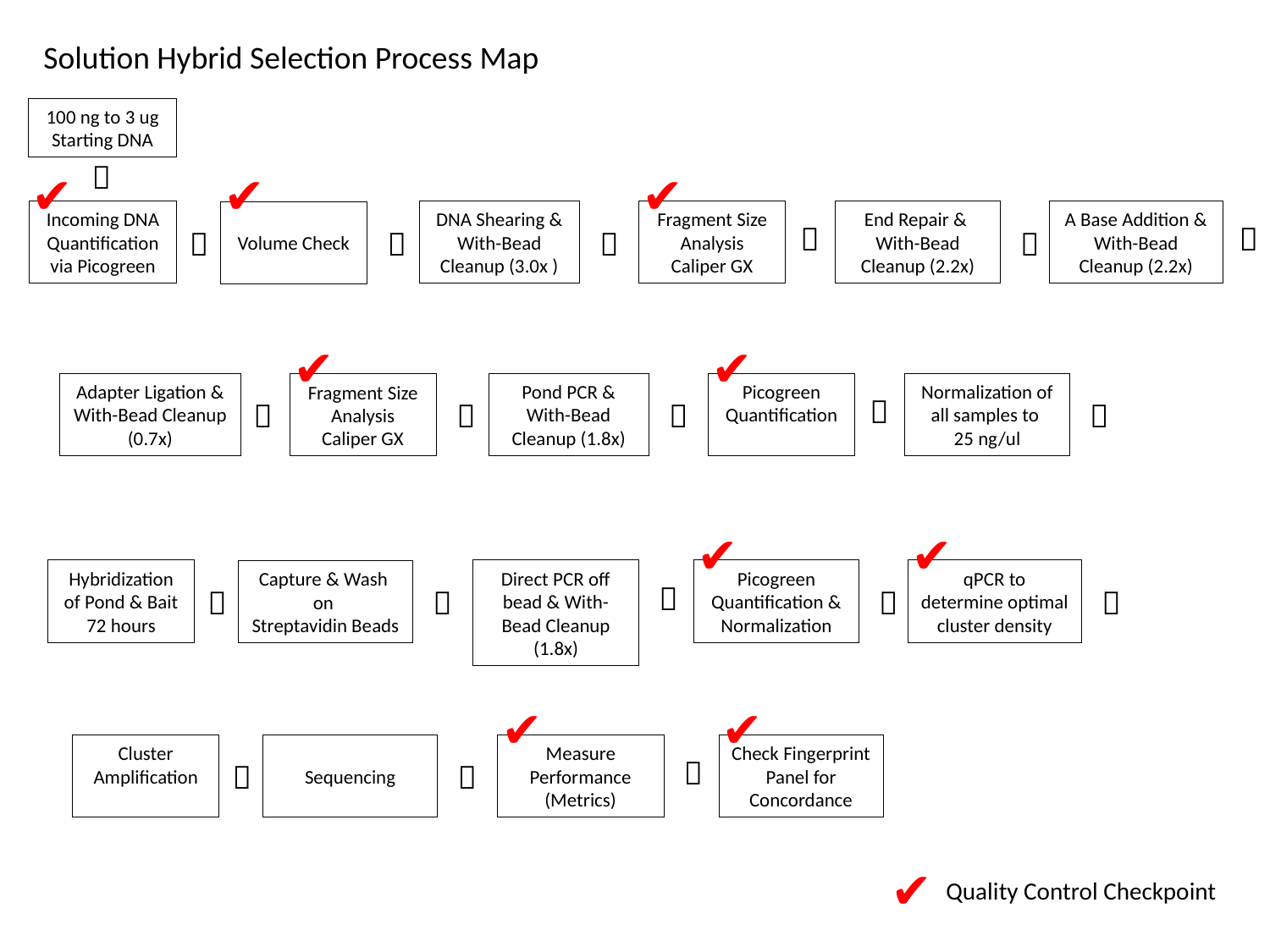

Solution Hybrid Selection Process Map
100 ng to 3 ug Starting DNA

✔
✔
✔
Incoming DNA Quantification via Picogreen
Volume Check
DNA Shearing & With-Bead Cleanup (3.0x )
Fragment Size Analysis Caliper GX
End Repair &
With-Bead Cleanup (2.2x)
A Base Addition & With-Bead Cleanup (2.2x)






✔
✔
Adapter Ligation & With-Bead Cleanup (0.7x)
Fragment Size Analysis Caliper GX
Pond PCR & With-Bead Cleanup (1.8x)
Picogreen Quantification
Normalization of all samples to
25 ng/ul





✔
✔
Hybridization of Pond & Bait
72 hours
Direct PCR off bead & With-Bead Cleanup (1.8x)
Picogreen Quantification & Normalization
qPCR to determine optimal cluster density
Capture & Wash
on
Streptavidin Beads





✔
✔
Cluster Amplification
Measure Performance (Metrics)
Check Fingerprint Panel for Concordance
Sequencing



✔
Quality Control Checkpoint
