# Supplementary material for: A scalable, fully automated process for construction of sequence-ready human exome targeted capture libraries
Source: Genome Biol. 2011 Jan 4;12(1):R1. doi: 10.1186/gb-2011-12-1-r1 (PMC3091298; doi:10.1186/gb-2011-12-1-r1)
Supplement: Additional file 4 — Manual SHS protocol. A word document outlining the manual protocol. [file gb-2011-12-1-r1-S4.DOCX]

**Manual Plate-Based “with-bead” Pond Library Construction, detailed protocol**

**DNA Shearing with the Covaris E210**

1. Begin with 3 ug of genomic DNA. Adjust this volume if necessary to 50 μl with TE. Transfer these samples to glass Covaris shearing tubes (Covaris; microTube snap-cap, cat. # 520045).
2. Fill bath level to 7.5 as marked on Covaris water bath container. Degas the water for approximately 40 minutes prior to shearing.
3. Shear DNA in glass Covaris tubes according to the following parameters (total volume = 50 μl); duty cycle = 20%; intensity = 5; cycles per burst = 200; time=165 seconds; z-height offset 6 mm.

**Post Shearing “with-bead” SPRI Cleanup**

1. Aliquot 150 of completely re-suspended AMPure XP SPRI (Beckman Coulter Genomics, cat. # A63881) beads into the wells of Eppendorf 96-well skirted plate (Eppendorf TwinTec 96 Plate, cat. # 47744116).
2. Transfer 50 μl of sheared DNA from each Covaris tube into corresponding plate well containing the SPRI beads.
3. Pipette mix full volume 15 times or cap plate and vortex. If vortexing the plate, flash spin to bring sample to well bottom.
4. Incubate at room temperature for 2 minutes.
5. Place the plate on MPC-96S plate magnet (Invitrogen, cat. # 12027) for 4 minutes. Remove supernatant and discard supernatant.
6. With new tips and with plate on the magnet, add 100 μl of 70% ethanol (without disturbing the beads).
7. Incubate 30 sec on plate magnet. Remove and discard ethanol (without disturbing the beads).
8. Remove any drops of ethanol from each well with pipette tip.
9. Remove plate from magnet. Let sample plate air dry for 2 minutes.
10. Add 40 μl of Tris-HCl (pH 8) to each well and pipette mix 15 times to re-suspend and elute DNA off of the beads.

**Shearing QC**

1. Take 3 μl of each sample and dilute with 12 μl TE buffer pH 8.0 run on Caliper Labchip DNA 1K Chip. Proceed to “Fragment End Repair” if sample fragments range between 75-300 base pairs, with the majority of fragments centered between 120-150 base pairs.

Note: Alternatively, Agilent’s DNA 1000 DNA (Agilent Technologies Inc, cat. # 5067-1504).

**“With-bead” Fragment End Repair**

1. Make master mix of End Repair reagents (refer to Table 1). Flick to mix and flash spin.
2. Add 27 μl of master mix to each well of plate, already containing DNA sample and mix with pipette 10 times.
3. Cap wells of plate with optical strip caps (Applied Biosystems, cat. # 4323032).

Table 1

End Repair Master Mix (New England Biolabs)

| Reagent | Volume (μl)/ rxn |
| --- | --- |
| 10X T4 DNA Ligase Buffer | 5 |
| BSA (1mg/ml) | 5 |
| ATP(10mM) | 5 |
| dNTPs (10mM) | 2 |
| T4 PNK | 5 |
| T4 POL | 5 |
| Mix Total Volume | 27 |

1. Run “End Repair” program on Eppendorf Thermal Cycler

“End Repair” program.

12°C 15 mins

25°C 15 mins

Total time 30 min

**Post “with-bead” Fragment End Repair Cleanup**

1. Dispense 147 μl of 20%PEG, 2.5M NaCl into the sample plate.
2. Pipette mix full volume 15 times or cap plate and vortex. If vortexing the plate, flash spin to bring sample to well bottom.
3. Incubate at room temperature for 2 minutes.
4. Place the plate on MPC-96S plate magnet (Invitrogen, cat. # 12027) for 4 minutes. Remove supernatant and discard supernatant.
5. With new tips and with plate on the magnet, add 100 μl of 70% ethanol (without disturbing the beads).
6. Incubate 30 sec on plate magnet. Remove and discard ethanol (without disturbing the beads).
7. Remove any drops of ethanol from each well with pipette tip.
8. Remove plate from magnet. Let sample plate air dry for 2 minutes
9. Add 40 μl of Tris-HCl (pH 8) to each well and pipette mix 15 times to re-suspend and elute DNA off of the beads.

**“With-bead” A-Base Addition**

1. Make master mix of A-Base Addition reagents according to Table 2. Flick to mix and flash spin.
2. Add 20 μl of master mix to each well of plate, already containing ~40 μl of DNA sample and mix with pipette 10 times.
3. Cap wells of plate with optical strip caps.

Table 2

A-Base Master Mix (New England Biolabs)

| Reagent | Volume (μl)/rxn |
| --- | --- |
| Nuclease Free Water | 2 |
| 10X Klenow Buffer | 5 |
| dATP (1mM) | 10 |
| Klenow exo- | 3 |
| Mix Total Volume | 20 |

1. Run “A-Base Addition” program on Eppendorf Thermal Cycler

“A-Base Addition” program

37°C 30 mins

Total time 30 min

**Post “with-bead” A-Base Addition Cleanup**

1. Dispense 132 μl of 20%PEG, 2.5M NaCl into the sample plate.
2. Pipette mix full volume 15 times or cap plate and vortex. If vortexing the plate, flash spin to bring sample to well bottom.
3. Incubate at room temperature for 2 minutes.
4. Place the plate on MPC-96S plate magnet (Invitrogen, cat. # 12027) for 4 minutes. Remove supernatant and discard supernatant.
5. With new tips and with plate on the magnet, add 100 μl of 70% ethanol (without disturbing the beads).
6. Incubate 30 sec on plate magnet. Remove and discard ethanol (without disturbing the beads).
7. Remove any drops of ethanol from each well with pipette tip.
8. Remove plate from magnet. Let sample plate air dry for 2 minutes
9. Add 40 μl of Tris-HCl (pH 8) to each well and pipette mix 15 times to re-suspend and elute DNA off of the beads.

**“With-bead” Adapter Ligation**

1. Mix 2x DNA Ligase Buffer and DNA Ligase according to Table 3. Flick to mix and flash spin. Place on ice until use.
2. With new tips, add 3 μl of Adapter Oligo mix to each sample.
3. Add 15.5 μl of 2X DNA Ligase Buffer and DNA Ligase Mix to each well of plate, already containing 40 μl of DNA sample.
4. Mix sample/Adapter Ligation components 10 times.
5. Cap wells of plate with optical strip caps.

Table 3

Adapter Ligation (New England Biolabs)

| Reagent | Volume (μl)/rxn |
| --- | --- |
| 2X DNA Ligase Buffer | 12.5 |
| DNA Ligase (1U/μl) | 2.5 |
| Mix Total sample/master mix volume | 15 |
| PE Adapter Oligo Mix (Illumina) | 3 |

1. Run “Adapter Ligation” program on Eppendorf Thermal Cycler

“Adapter Ligation” program

25°C 15 mins

Total time 15 min

**Post Adapter Ligation “with-bead” Cleanup**

1. Dispense 40 μl of 20% PEG, 2.5M NaCl into the sample plate.
2. Pipette mix full volume 15 times or cap plate and vortex. If vortexing the plate, flash spin to bring sample to well bottom.
3. Incubate at room temperature for 2 minutes.
4. Place the plate on MPC-96S plate magnet (Invitrogen, cat. # 12027) for 4 minutes. Remove supernatant and discard supernatant.
5. With new tips and with plate on the magnet, add 100 μl of 70% ethanol (without disturbing the beads).
6. Incubate 30 sec on plate magnet. Remove and discard ethanol (without disturbing the beads).
7. Remove any drops of ethanol from each well with pipette tip.
8. Remove plate from magnet. Let sample plate air dry for 2 minutes
9. Add 40 μl of Tris-HCl (pH 8) to each well and pipette mix 15 times to re-suspend and elute DNA off of the beads.
10. Place the sample plate on the magnet for 3 minutes.
11. Transfer supernatant to a new 96 well-plate. These samples are now referred to as “ponds.”

**Pond PCR Enrichment**

1. Make master mix of PCR reagents in a 1.5 ml tube. Flick to mix and flash spin tube in microfuge.
2. Add 20 μl of master mix to each well of the sample plate, already containing 40 μl of DNA sample and mix with pipette 15 times.

Table 4

Pond PCR Enrichment master mix

| Reagent | Volume (μL/rxn |
| --- | --- |
| Omnipure Water (VWR; PAP 1193) | 7 |
| 10x PFU Buffer (Agilent Technologies - Stratagene Products, cat. # 200532 | 6 |
| 10mM dNTPs (400ul @ 100mM; Agilent Technologies, cat. # 200415) | 1 |
| PFU enzyme (Agilent Technologies, cat. # 929674) | 2 |
| PCR Primer PE 1.0 (Illumina; Component of Paried-End Sample Prep Kit v1,Vendor cat. #PE-102-1001) | 2 |
| PCR Primer PE 2.0 (Illumina; Component of Paried-End Sample Prep Kit v1,Vendor cat. #PE-102-1001) | 2 |
| Total Volume | 20 |

1. Cap wells of plate with optical strip caps.
2. Run Pond PCR program on Eppendorf Thermal Cycler.

“Pond/Catch PCR” program:

95°C 2 min’

95°C 30 sec

6X

65°C 30 min

72°C 1 min

72°C 10 min

4°C hold

**Post Pond PCR SPRI Cleanup**

1. Dispense 90 μl of completely re-suspended SPRI beads into the sample plate.
2. Pipette mix full volume 15 times or cap plate and vortex. If vortexing the plate, flash spin to bring sample to well bottom.
3. Incubate at room temperature for 2 minutes.
4. Place the plate on MPC-96S plate magnet for 4 minutes. Remove supernatant and discard supernatant.
5. With new tips and while plate is on the magnet, add 100 μl of 70% ethanol (without disturbing the beads).
6. Incubate 30 sec on plate magnet. Remove and discard ethanol (without disturbing the beads).
7. Remove any drops of ethanol from each well with pipette tip.
8. Remove plate from magnet. Let sample plate air dry for 2 minutes.
9. Add 40 μl of Tris-HCl (pH 8) to each well and pipette mix 15 times to re-suspend and elute DNA off of the beads.
10. Place the sample plate on the magnet for 3 minutes.
11. Transfer supernatant to a new plate. These samples are now referred to as “enriched ponds.”
12. Normalize your “enriched ponds” to 25 ng/μl before proceeding to the Manual Hybridization Setup. 20 μl at a concentration of 25ng/μl is required for hybridization.

Note: If storing samples, cap plate and store at -20°C.

**Manual Hybridization Setup**

1. Thaw the following reagents on ice: 50X Denhardt’s Buffer (Sigma Alrich CO, cat. # D2532), Human Cot-1 DNA (1 mg/ml, Invitrogen, cat. # 15279101), Salmon Sperm DNA (5x10mg/ml, Invitrogen, cat. # 15632-011), and SureSelect Block#3 (from Sure Select Target Enrichment System, Agilent Technologies).
2. Turn on plate compatible heat block and set to 65°C.
3. Allow aliquots of baits (Component of SureSelect Human All Exon Kit, Agilent Technologies) to thaw on ice. Also place normalized, enriched ponds on ice.
4. Obtain three, 96-well plates. Label one as “HYBRIDIZATION BUFFER”, one as “BAIT’ and one as “TARGET”.
5. Determine which wells will be used for sample and mark the corresponding wells on all three plates for easy identification during reagent aliquoting.
6. Prepare the Hybridization buffer, as described in Table 5 (page 12). Place hybridization buffer on heat block for at least 15 minutes.
7. Dilute the Salmon Sperm DNA 1:10 with Nuclease Free Water.
8. Prepare the carrier DNA-blocking Agent mixture according to Table 6 (page 12).
9. Set two thermocyclers to hold at block temperature of 65°C and the lid temperature of 105°C.
10. Prepare a third (non-incubating) thermocycler, by ensuring the following thermoprofile has been entered: 95°C for 5 minutes, hold at 65°C hold. Name the cycling protocol Hybridization.
11. Aliquot 40 μl of the hybridization buffer in each well of the HYBRIDIZATION PLATE.
12. Cap the wells of the HYBRIDIZATION BUFFER and place on the thermocycler holding at 65°C.
13. Gently vortex and spin down your normalized, enriched ponds. Aliquot 20 μl of each pond into marked wells of the TARGET plate. Cap wells and place on ice.
14. Aliquot 8 μl of the carrier DNA-Blocking Agent into wells on the TARGET plate containing samples.
15. Add 1 μl of Superase Rnase inhibitor (20U/ul, Applied Biosystems, cat. # AM2694) for every 5 μl of bait into bait tube. Be sure to pipette mix gently. NOTE: Bait should be at a concentration of 100 ng/ul.
16. If spiking in control bait (see main text), add 1 ul at a concentration of 360 pg/ul.
17. If NOT spiking in control bait, Aliquot 6 μl of the Superase/Bait mixture into designated well of BAIT plate. Cap and place on ice. IF spiking in control bait, add 7μl of the Superase/Bait/control bait mixture into designated well of BAIT plate.
18. Place the TARGET plate on the third (non-incubating) thermocycler, and start the hybridization protocol.
19. Monitor the time of the hybridization protocol on the thermocycler. When there are two minutes remaining of the 95°C denature step of the Hybridization program, plate the BAIT plate on the remaining thermocycler set to 65°C and close the lid.
20. When the time remaining on the 95°C denature step of the Hybridization program reaches approximately 2 seconds, pause the protocol (so the block holds at 95°C) and open the lid of the thermocycler and remove the Optical Caps.
21. Immediately open the lids of the thermocyclers containing the HYBRIDIZATION BUFFER plate and the BAITS plate and quickly remove the Optical Caps.
22. Quickly but carefully transfer 6 μl of the Superase-Bait mixture from the BAITS plate into the TARGET Plate. Pipette mix 3 times.

NOTE: Be sure the Superase-Bait mixture is being added to well of the TARGET plate that contain sample and hybridization buffer.

1. Quickly but carefully transfer 34 μl of the hybridization buffer from the HYBRIDIZATION BUFFER plate into the TARGET Plate. Pipette mix 3 times.

NOTE: Be sure the hybridization buffer is being added to wells of the TARGET PLATE that contain sample.

1. Cap the wells of the TARGET plate using ABI Optical Caps, close the lid of the thermocycler and resume the hybridization cycling program.
2. Allow the hybridization program to continue running at 65°C for 72 hours.

NOTE: Agilent’s SureSelect protocol requires a 21 hour hybridization. Our current production protocol requires a 72 hour hybridization. Samples used to generate data presented in this paper were hybridized for 72 hours.

1. After the TARGET plate has incubated at 65°C for 72 hours, remove the TARGET plate and proceed to the Manual Capture Protocol.

**Manual Capture Protocol**

Note: It is recommended that samples be processed in 1.5ml RNA-ase free microfuge tubes (Ambion, cat. # AM12450) at this point, as portions of this protocol require constant mixing for .5 hours. This is easily accomplished by placing sample tubes a rotor.

1. Set two (1.5ml tube or 96-well plate compatible) heat blocks to 65°C.
2. Prepare the following Buffers in 50ml conical tubes (Corning, cat. # 430921) according to List A.

NOTE: Volumes may be scaled down to accommodate smaller sample sizes

List A

BW Buffer

A) 40 ml nuclease-free water

B) 10 ml 5M NaCl

C) 50 μl 1M Tris-HCl (stored at +4)

D) 100 μl 0.5M EDTA

AP Buffer

A) 40 ml nuclease-free water

B) 2.5 ml 20x SSC

C) 500 μl 10% SDS

GS Buffer (store at 65^o^C)

A) 49 ml nuclease-free water

B) 250 μl 20x SSC

C) 500 μl 10% SDS

1. Shake M-280 Streptavidin coated Dynabead (Invitrogen, cat. # 112-05D) vial to obtain a homogeneous suspension.
2. For every sample to be captured, transfer 50 μl of Dynabeads to a 15 ml conical tube.

NOTE: It’s recommended to add four additional samples worth of beads for dead volume).

1. Add the appropriate volume (200 μl of BW for every 50 μl of beads) to the15 ml tube containing beads.
2. Gently vortex and place tube in DynaMag-15 (Invitrogen, cat. # 123-01D) bead separator for 1-2 minutes.
3. Remove and discard supernatant without disturbing the attached beads.
4. Repeat steps 104 through 106 two additional times.
5. Resuspend the DYNAl M-280 beads in 100 μl BW buffer per 50 μl beads initially aliquoted into the 15 ml tube. Vortex to achieve a homogenous mixture. Once the DYNAl M-280 have been resuspended, the content of each conical tube may be combined to ensure a homogenous mixture of washed M-280 beads.

!CAUTION!: Incorrect concentration of M-280 Dynabeads may result in low catch product yield

1. If samples are being captured in a plate, aliquot 102 μl of washed beads into each well of an Eppendorf 96 well skirted plate (beads location in a plate should match that of the sample location in the Hybridization plate). If samples are being processed in tubes, leave the beads in the 15 ml conical tube (BD Product Number: 352098).
2. Store M-280 beads in 15 ml tube or plate at 4^o^C until use.
3. If samples are being captured in a plate, aliquot 2 ml GS buffer into a Costar 96-Deep well plate (GS buffer location in a plate should match that of the sample location in the Hybridization plate). Place on a heat block at 65°C. If samples are being processed in tubes, leave the GS in the 50 ml conical tube and incubate this at 65°C.
4. If samples are being captured in a plate, aliquot 180 μl of AP buffer into a 96-well plate (AP buffer location in a plate should match that of the sample location in the Hybridization plate). If samples are being processed in tubes, leave the beads in the 15 ml conical tub

**Sample Binding/Bead Addition**

1. Remove your TARGET plate from the thermocycler.

Note: It is recommended that samples be TRANSFERED from a plate into 1.5ml RNA-ase free microfuge tubes (Ambion, cat. # AM12450) at this point, as portions of this protocol require constant mixing for .5 hours.

!Caution: begin protocol as soon as possible after the TARGET Plate is removed from the thermocycler!

1. Remove M-280 beads from storage at 4°C.
2. Add 100 ul of washed beads to each sample (tube or well).
3. If samples are in a tube, rotate to mix for 30 minutes. If samples are in a plate, mix approximately 120 μl, 200 times.
4. Resuspend settled beads and place sample tubes on a DynaMag-2 (Invitrogen, cat. # 123-21D) magnet or TARGET plate on a MPC-96S plate magnet (cat. # 120-27). Let the sample tubes or TARGET plate to sit on the magnet plate for approximately 2 minutes.
5. Remove supernatant and discard waste and tips.
6. Take sample tubes/Plate off of the magnet.
7. Add 180 μl of AP Buffer to sample well/plate.
8. Mix full volume 10 times.
9. Incubate sample with AP buffer at room temperature for 15 minutes.
10. Mix 75 μl of sample an additional 15 times.
11. Place sample tubes on a DynaMag-2 tube magnet or the TARGET plate Dynal MPC-96S plate magnet for 2 minutes. This will allow the M-280 streptavidin beads to separate from the AP buffer.
12. Remove and discarded supernatant and tips.

**GS Wash**

1. Add 180 μl of GS Buffer (at 65°C) to sample tubes/plate. Mix ten times.
2. Place the sample tubes/ plate on the 65°C heat block for 10 minutes.
3. Keeping the sample tubes or TARGET plate on the 65°C heat block, perform a Dual Height Mix. Mix 120 μl, 15 times.
4. Allow the sample tubes or TARGET plate to sit on the heat block and incubate at 65°C for 10 minutes.
5. Keeping the sample tubes or TARGET plate on the heat block, mix 75 μl of sample an additional 15 times.
6. Place sample tubes/plate on a Dynamag™-2 tube magnet for 2 minutes.
7. Remove supernatant and discard into the waste.
8. Repeat steps 126-132, five additional times. DNA “catches” are on the dry M-280 beads.

**“Off-bead” Catch Enrichment**

1. Prepare the appropriate amount of master mix according to the Table 7 (page 12).
2. Aliquot approximately 50 μl of Catch Enrichment master mix to the sample tubes/ plate and mix ten times.
3. If not already in a plate, transfer beads/PCR master mix to a 96-well plate and cap the wells.
4. Place in thermocycler and begin Catch Enrichment PCR Program.

“Pond/Catch PCR” program:

95°C 1 min’

95°C 30 sec

20X

65°C 30 min

72°C 1 min

72°C 10 min

4°C hold

**Post “off-bead” Catch Enrichment Cleanup**

1. Mix sample wells until beads are resuspended.
2. Place the plate on the Dynal MPC 96-S plate magnet plate for 5 minutes.
3. Aliquot 90 μl of completely re-suspended AMPure XP SPRI beads into a new 96-well plate.
4. Transfer supernatant from catch enrichment plate from the sample plate, into 96-well plate containing the AMPure XP SPRI beads.
5. Pipette mix full volume 15 times or cap plate and vortex. If vortexing the plate, flash spin to bring sample to well bottom.
6. Incubate at room temperature for 2 minutes.
7. Place the plate on MPC-96S plate magnet for 4 minutes. Remove supernatant and discard supernatant.
8. With new tips and while plate is on the magnet, add 100 μl of 70% ethanol (without disturbing the beads).
9. Incubate 30 sec on plate magnet. Remove and discard ethanol (without disturbing the beads).
10. Remove any drops of ethanol from each well with pipette tip.
11. Remove plate from magnet. Let sample plate air dry for 2 minutes
12. Add 40 μl of Tris-HCl (pH 8) to each well and pipette mix 15 times to re-suspend and elute DNA off of the beads.
13. Place the sample plate on the magnet for 3 minutes.
14. Transfer supernatant to a new 96-well plate. These samples are now referred to as “ enriched catches.”
15. Store samples at -20 °C until sequencing.

Table 5

Hybridization Buffer


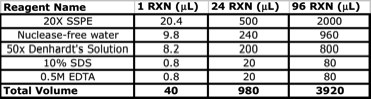


Table 6

Carrier DNA-blocking agent mix


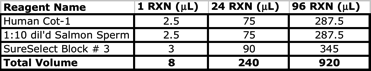


Table 7

“Off Bead” Catch Enrichment Master Mix
