# Supplementary figures and images for: A scalable, fully automated process for construction of sequence-ready human exome targeted capture libraries
Source: Genome Biol. 2011 Jan 4;12(1):R1. doi: 10.1186/gb-2011-12-1-r1 (PMC3091298; doi:10.1186/gb-2011-12-1-r1)

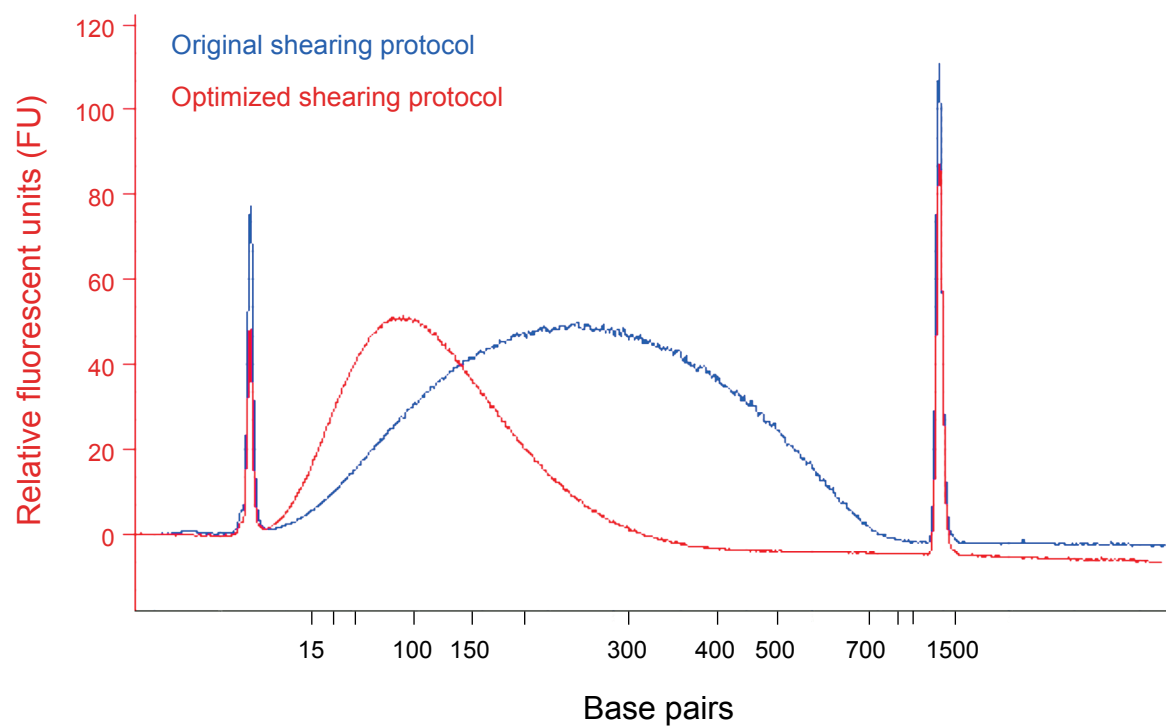

Supplement: Additional file 5 — DNA shearing optimization. Profiles of sheared genomic DNA from unoptimized (blue) and optimized (red) conditions are shown. The size distribution from optimized conditions has a larger fraction of product DNA in the desired size range of 120 to 150 bases. The sharp peaks at approximately 20 and approximately 1,500 bases represent size standards. [file gb-2011-12-1-r1-S5.PDF]

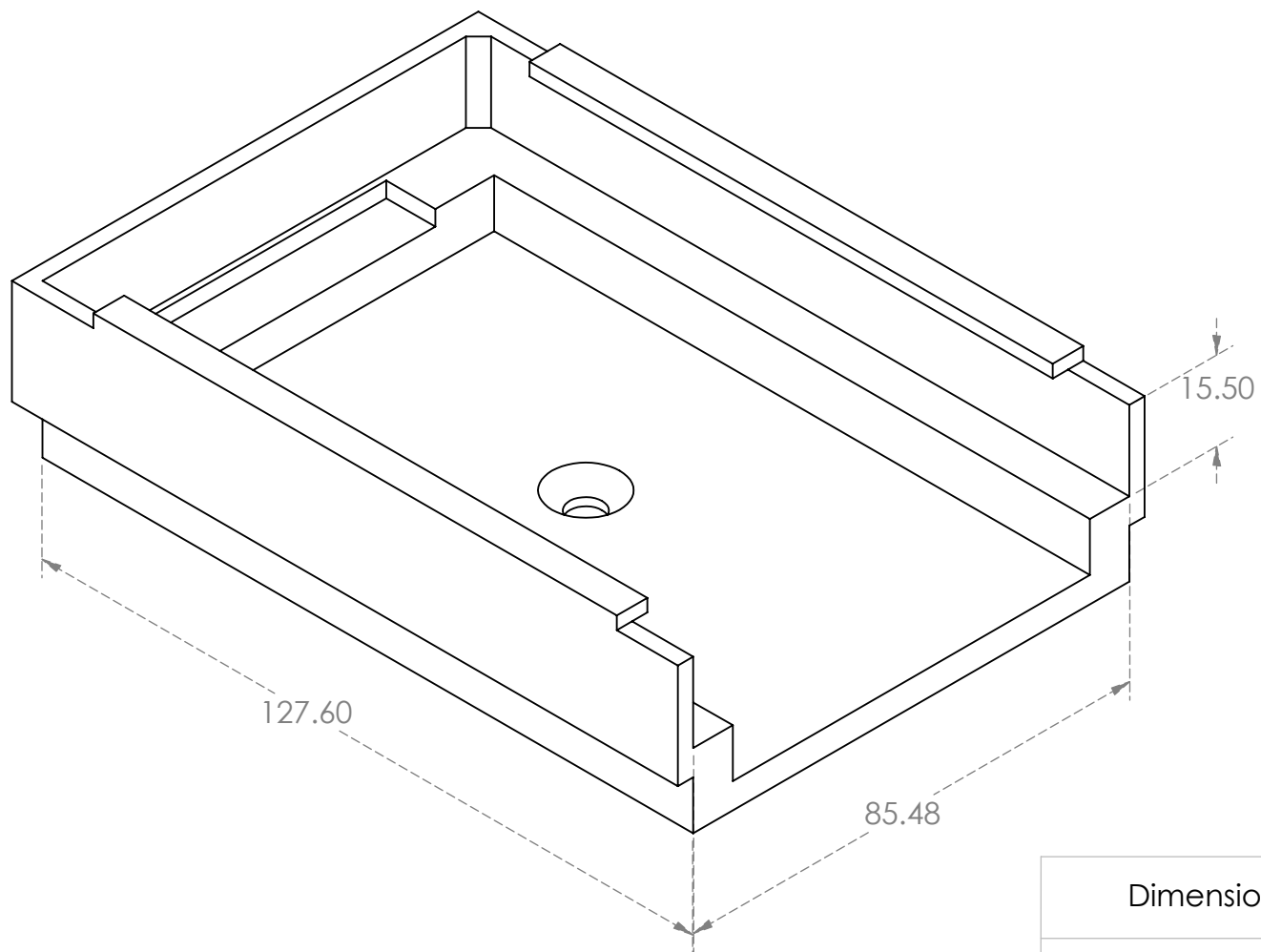

Dimension (mm)

05/05/2010

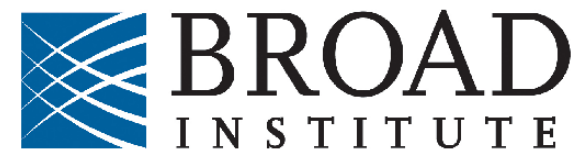

TITLE: Agilent Bravo:  
Holder for Covaris Rack Transfer

WEIGHT:

SHEET 1 OF 1

Supplement: Additional file 6 — Shearing rack CAD drawing. A PDF showing the CAD drawing and dimensions for the shearing rack adapter for the Covaris unit. [file gb-2011-12-1-r1-S6.PDF]

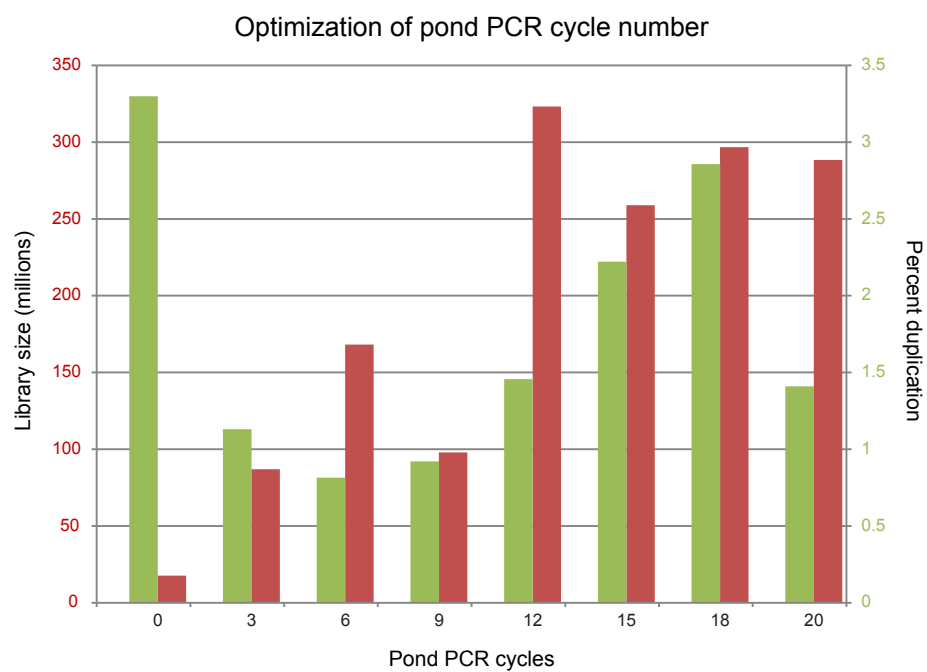

Supplement: Additional file 7 — Optimization of pond PCR cycle number. For each number of PCR cycles tested, red bars (left-hand y-axis) show number of unique molecules per library, in millions; green bars (right-hand y-axis) show percent duplicated sequences. Data were generated in a controlled experiment using high quality human female DNA purchased from Promega (Madison WI, USA, catalogue number G1521). Patient samples typically demonstrate lower performance likely due to lower sample quality. [file gb-2011-12-1-r1-S7.PDF]

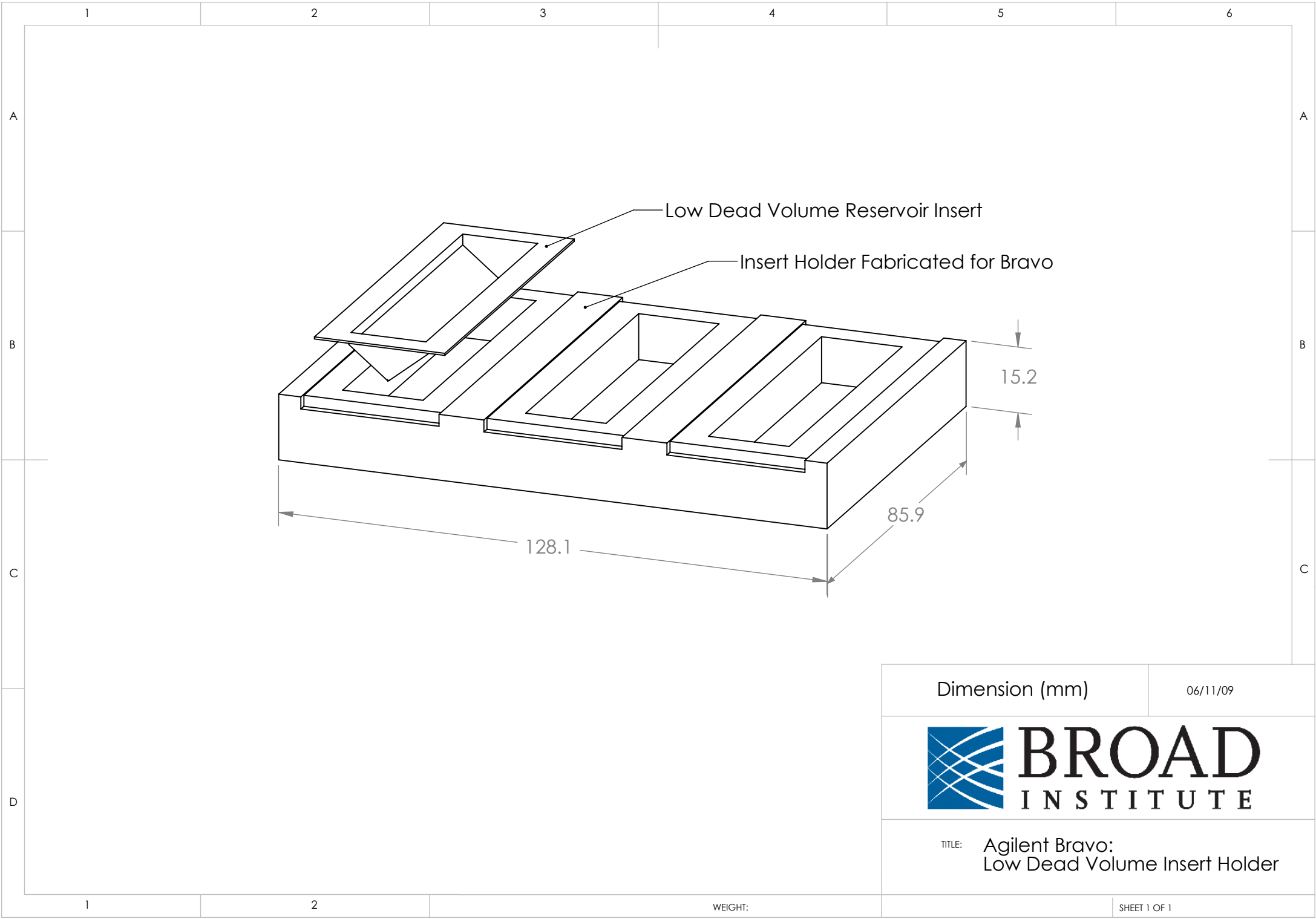

|                                                                                                                 |          |
|-----------------------------------------------------------------------------------------------------------------|----------|
| Dimension (mm)                                                                                                  | 06/11/09 |
| 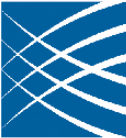 <b>BROAD</b><br>INSTITUTE |          |
| TITLE: Agilent Bravo:<br>Low Dead Volume Insert Holder                                                          |          |

Supplement: Additional file 8 — Reagent reservoir CAD drawing. A PDF showing the CAD drawing and dimensions for the low volume custom reservoir used for reagent dispensing. [file gb-2011-12-1-r1-S8.PDF]

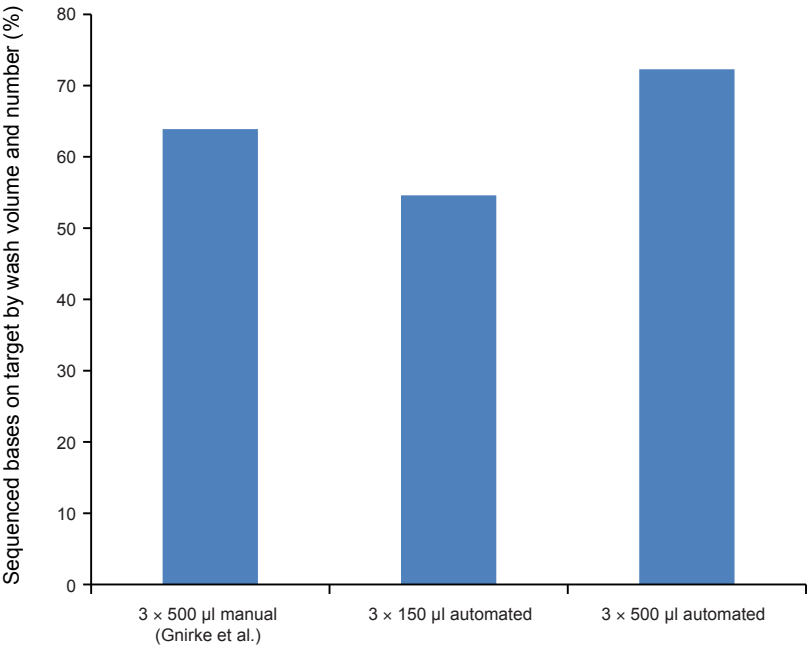

Supplement: Additional file 9 — Optimization of hybrid selection wash conditions. Results for three sets of conditions are shown: manual protocol from Gnirke et al. [14], with three 500-μl washes; unoptimized automated protocol, with three 150-μl washes; optimized automated protocol, with six 150-μl washes. Shown are percent sequenced bases on target for a controlled bait set. [file gb-2011-12-1-r1-S9.PDF]

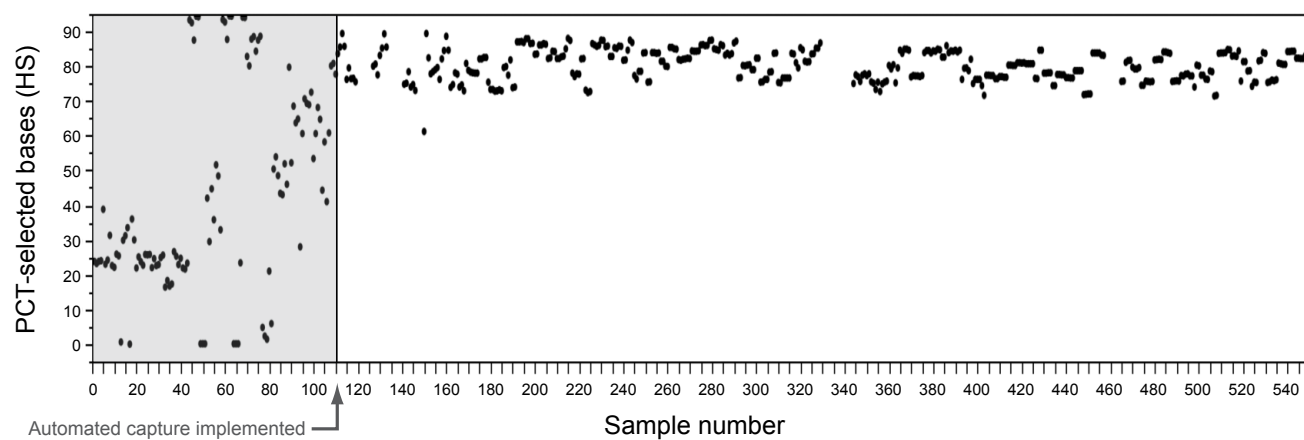

Supplement: Additional file 10 — Improved process control with transition from manual to automated capture. Implementation of the automated capture protocol greatly reduced sample to sample variability as measured by the percent of bases on or near the target. Data from 550 samples from the production process are shown. Samples in the gray box (the first 110) were performed manually, and samples on the white background represent the first group run with the automated protocol. [file gb-2011-12-1-r1-S10.PDF]

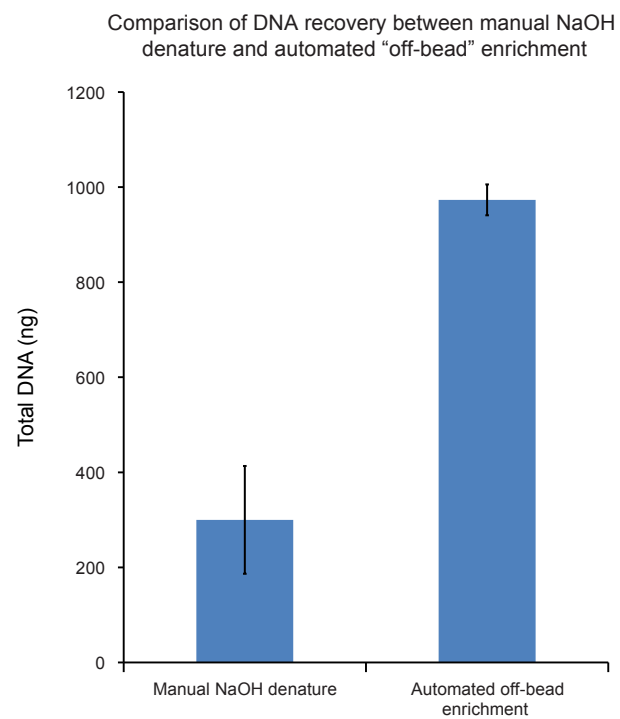

Supplement: Additional file 11 — Comparison of DNA recovery between manual NaOH denaturation and automated 'off-bead' enrichment. Total yield of DNA in nanograms is shown. [file gb-2011-12-1-r1-S11.PDF]
