# Supplementary material for: A scalable, fully automated process for construction of sequence-ready human exome targeted capture libraries
Source: Genome Biol. 2011 Jan 4;12(1):R1. doi: 10.1186/gb-2011-12-1-r1 (PMC3091298; doi:10.1186/gb-2011-12-1-r1)
Supplement: Additional file 12 — Sequencing metrics definitions. A Word document that defines the sequencing metrics used to measure process performance. [file gb-2011-12-1-r1-S12.DOCX]

**Sequencing Metric Definitions**

We use a number of key metrics to evaluate the sequence data derived from our SHS libraries. All the metrics described here are implemented as part of the CalculateHsMetrics program available as part of the Picard [31, 32] suite of programs in both executable and source forms.

For the following calculations, these terms are defined: Duplicated molecules are defined as passing read pairs where both reads have identical start sites to both reads in another read pair. Unique reads are thus defined as those read pairs that are not duplicated.

ZERO_CVG_TARGETS_PCT: the number of targets receiving no significant coverage. Since the selection process and alignment algorithms are imperfect it is common to observe untargeted regions of the genome with a single read mapped to them. Because of this we define a target as “zero” coverage if across its entire length no single base is covered by two or more reads.

MEAN_TARGET_COVERAGE: the mean number of reads covering each target base computed across all bases in non-zero coverage targets. For the purpose of this calculation, only reads from unique molecules are counted so as not to inflate coverage due to duplication.

FOLD_80_BASE_PENALTY: a measure of the non-uniformity of sequence coverage: the amount of additional sequencing that would be necessary to ensure that 80% of target bases (in non-zero coverage targets) are covered to the current mean target coverage. This is calculated by taking coverage at the 20^th^ percentile and dividing by the mean target coverage.

PCT_TARGET_BASES_2X: The percentage of all target bases that are covered by 2 or more reads from unique molecules.

PCT_TARGET_BASES_10X: The percentage of all target bases that are covered by 10 or more reads from unique molecules.

PCT_TARGET_BASES_20X: The percentage of all target bases that are covered by 20 or more reads from unique molecules.

HS_LIBRARY_SIZE: The estimated number of unique construct molecules in the SHS library that will map to the target regions. This is calculated from the sum of read pairs observed on or near the targets and the number of unique read pairs and then solving the following formula numerically as in Lander and Waterman [Lander]:

U/Ls = 1 - exp(-N/Ls)

where Ls = the estimated number of unique molecules in the library

N = the number of read pairs observed

U = the number of unique read pairs observed

We solve computationally for Ls by initializing Ls to U and then repeatedly carry out these three steps until Ls is defined: 1) calculate both sides of the equation, 2) compare values, and if they are equal they define Ls, 3) increment or decrement Ls by a heuristically determined factor and repeat.

PERCENT_DUPLICATION: The percentage of mapped sequence reads that are marked as originating from duplicate molecules.

PCT_SELECTED_BASES: The percentage of mapped bases from sequence reads that are mapped to regions within 250b of the target regions.
