# Supplementary material for: A scalable, fully automated process for construction of sequence-ready human exome targeted capture libraries
Source: Genome Biol. 2011 Jan 4;12(1):R1. doi: 10.1186/gb-2011-12-1-r1 (PMC3091298; doi:10.1186/gb-2011-12-1-r1)
Supplement: Additional file 13 — Fingerprint bait sequences. A Word document listing the sequences of baits used in the fingerprint panel. [file gb-2011-12-1-r1-S13.DOCX]

**Supplementary Table 3. Fingerprint Panel Bait Sequences**

| >rs1210110 | atcgcaccagcgtgtTGACATTTAGTATCAGCTAGGTGCTCTAACATTCTTTTAGGTAGGGAATAAAACTTAAAATAGCACTTATGTTTTAGACAGTTATGTGATTTCATTTTGAGTGCATCTTAATGGTATTAAGATTGTACTTCCTTTTGTTAcactgcggctcctca |
| --- | --- |
| >rs7555566 | atcgcaccagcgtgtGGATGGATAGAAGGAAGGAAGGAAGGAAGAAAGGATGACAAGTGAATACACAGATGGAAGAATGAATCTATCTAGGTGGATGGAAAAAAGGATAAATGAAAGGTTGGACGGATGGGTGGATGGCAGTTTTGGACACTTGAcactgcggctcctca |
| >rs1364054 | atcgcaccagcgtgtCTTCAAGTCAGGCTGGGGACAGGATCAATGGCCTCAGTGGTTAGGAACAATGACATCAATGTGCTAATTATCTCTGTGATTCTGAGTGTAAATGCTCTGGTCCCTACAGTCCATAGGCATGACAGATGATGACTAATGGGcactgcggctcctca |
| >rs6734275 | atcgcaccagcgtgtCACTTTCAAAATAACAAAGACACTGAGAGGCTAAGTATCTTGCCCAAGGTTACACAGTTGGTAAGATACGGGATGTAAACACAGCCAGTCTGTTTCCAGAGTCTGCATTCCATAGAGTGTGGTATAATGTATTATAAAATcactgcggctcctca |
| >rs7584993 | atcgcaccagcgtgtCACACACACACACACACACTGGTAAGTAGGTGAGGTGATGGATGTATGAACAAAATTGATTGTGGTCATCATTTTTTCACAATATATACATATATCAAATCCTAATATTGTACATCTTAAACTGATAGAGTTTTGTCAGTcactgcggctcctca |
| >rs17272796 | atcgcaccagcgtgtAGAAGTTTATATCTTATCCAATCTGGCCCTCACTCCTCTTCTATCCATGTTTCCTTACATTCTCTAATACGAGTTGAGGTGCCTAGAAAGCTTAACAATATTTTACATGATTTCAAGTCCTGGCCTTCCACATCATGCCTcactgcggctcctca |
| >rs1155741 | atcgcaccagcgtgtTCAAGTACAACATTTTCTCTAGCAATTATGGGGGTTTTATCTCCCAGATGTGATTTATGCCATATTACATTCCAAGAAGGGAACATGAAGAATGTTTGTGGCTAAAGAAGTCTCAACACAAGGAAACTACAGGTGAAACGcactgcggctcctca |
| >rs161792 | atcgcaccagcgtgtGAAGAAATTCAGAGAAAAAAACACACTGTATCACTTAGGAATTGACTCTGGCTGCTAGGAACAGAGAACTGACTATAGTGACTAAAAATGTGGGCATTTTATTCTTTCATTAATGGAAGTCCAGAAGTAGGCCCTCCAGGcactgcggctcctca |
| >rs11940551 | atcgcaccagcgtgtAAGGGGCTCTTCCTTTCATGGAATTACGGTCGCATCTATGGAAAAATTGAGCCAAATCCGAGATAGGTTTATAAAGAGATTACATTGATTTTCTACTCTCTGTGATTCTCTCTTCTTTCTCCATGTTGTGGGAGTCCTACcactgcggctcctca |
| >rs9293511 | atcgcaccagcgtgtCATTACAATGACCTTGAAGTCACAATATTAAAGGAAAAAGATAAAAGAAAGCTGGAAAAACTGGAAGAGCGCTTACTATTCTGCGCGCAGAAAATATTTAATTTAAACACTCTAAGAAATTACGTCCAAAGAATCATTATcactgcggctcctca |
| >rs9352613 | atcgcaccagcgtgtATTGCAAAGGAAATGTTTAATTGTAAAGAGATAACTGTTTTTTTGTACATGTGTTCCAACAGGAGATTCATGAAAACTTAACTGAACTTAACATGGTTATATGAGACAGCAAGTGACATGAAGGAGCAGACCACCAAGATcactgcggctcctca |
| >rs685449 | atcgcaccagcgtgtTGTTTAGGAGAAACAAAAAAGACAAAAAGAGGCTGGGATGGAAAAGAGAAGACATTCTAGGAACCTCTATGTTCTCTAAGTGCTTTGAAGCCCTCTGGAGCTCCATCACACAGGGCTCCAAGGGCCTGGCCACATTATTAcactgcggctcctca |
| >rs7808249 | atcgcaccagcgtgtACTTCTGCTTATGGGAAGCCCATTTAGATAACCTGGACATCAAACCTGATGAAAAAAGTGCTTGGATTTAACTGGACCTAGGAAGAGTATATTCAAGGTTATGATTATAGCATAGGATTACCTCACTCTTAGGAACAGCAcactgcggctcctca |
| >rs1106334 | atcgcaccagcgtgtAAAATTCCCCAGTGGGCTATTGGGAATCATGTTGAAAGGGGCCATTCCTGCTTCCATCCCTTGAGTCCCAGATCCACATATTCTCTGATTAAGGACACAGTGCCATTTAGAGGTTTCTGATTTAGTGCATAAGTTGAATCcactgcggctcctca |
| >rs11017876 | atcgcaccagcgtgtGGGAAAAGAGCCAAGTCAGGCGCCCAGTTTCTACATAAATGGCACCCCACAGCCTCTCTTCACCCTACGGTTCCTTTTCCCTGGCTCCAAGGCTGCTCCGACAGACGGGCTTCGGGCCACAGCTGGCAGCAGGAGAGCAAcactgcggctcctca |
| >rs9572094 | atcgcaccagcgtgtCAGGACCACTTTTCTGGAAAATCACCAGTTCAACAACTCTTAAAAAACTTCCAACGGGAGATGAGAAATTTACCTCTCCCAATAGTCTAGCCACCCGCCATCTTTGGGACTAGCATTCTGACTTATACCAGGCTGGAAGAcactgcggctcctca |
| >rs4905366 | atcgcaccagcgtgtTGAGAGAAGCACGTAGGTAATTGCCTGCTGAAGGTAGAGGGAAGAGGAGCAGGAACCTTTCACCTCACCATCTGCTCAGGCTCAGCCCCATCCCTGCAGAATCCTGAGCAGGGGCTCCACTCTCCTCTCTTCCTTGCACCcactgcggctcctca |
| >rs4775699 | atcgcaccagcgtgtTTTACATTGTGCAATTTCATGTGACATAGTGATTTTTAGGAACACATATGTCACCTTATGGCAGAACTACATATAAACAAGCAAACTGCCAAATAAACAAGATAATTCCATGTAATGGGAAGTAGTTTGAGAAAAGTAAAcactgcggctcctca |
| >rs1528601 | atcgcaccagcgtgtTGAAAAACAATAAAATTGTGCTAAAGTGATGCTATGCAAGGATACTTAATAGCACTAAGGAATGTTTATGTTATGGTGTTAGTAATAATACTAAAAAGTATAAATAATATTTATTGAGTGCTATTACATGCAGCCACTCTcactgcggctcctca |
| >rs11655512 | atcgcaccagcgtgtAACTACTGAACCCAAGTGATCCTCCTGCCTCAGCCTCCTGAGTAGCTGGGACTACACATCTACCCAGCAAGATCATTATAATCACACCTGTACCCTCTGGTCACCAAAGGATGTGAATTTTAAGTGGAGGTAGAGGGAGAcactgcggctcctca |
| >rs4793172 | atcgcaccagcgtgtACCTGAGTCCCTGAAAGCTTTCTTCCTGACTTCAACAGTCAAGGAGACCTACTGCCATCACCCAATAAAAAGGATTCCATCTTGTGTCCAAAAGGGCCATTTGGTACAAACACCCTGCCAGTATTTGAATCTCTTCCACAcactgcggctcctca |
| >rs242076 | atcgcaccagcgtgtTAATGACAACACTTACTTTATAGGGCCCTAGTGAGGCTCAAATGGGAAAATCCACGGAAAGCATTTAGCACTGATCATGCATGGAAAGTGCCTGATAGCTGATGGCACCTATTGTAACTAATAATAGTATTAGAATTAGTcactgcggctcctca |
| >rs6603251 | atcgcaccagcgtgtATAATTGCTCATATTAAAAGCTAAAATAACAATAAGAGGATAAACCAAAGCTATGGAAACTATTACCTCTGGGGTAGAAACAGCACAGACAGGGACAGAACCTGGACTTACCTGAATGTGTCTAGTTTGTTAGTGCTCACcactgcggctcctca |
| >AMG_mid100 | atcgcaccagcgtgtCAACCATCAGAGCTTAAACTGGGAAGCTGATGGTAGGAACTGTAAAATTGGGACCACTTGAGAAACCACTTTATTTGGGATGAAGAATCCACCCACTATTCTTTACAGAGCCCAGGGGACTGCTAATGCAAACAGTGATCcactgcggctcctca |
