# Supplementary material for: A scalable, fully automated process for construction of sequence-ready human exome targeted capture libraries
Source: Genome Biol. 2011 Jan 4;12(1):R1. doi: 10.1186/gb-2011-12-1-r1 (PMC3091298; doi:10.1186/gb-2011-12-1-r1)
Supplement: Additional file 14 — Automated SHS library construction protocol. A Word document detailing the automated SHS library construction protocol. [file gb-2011-12-1-r1-S14.DOCX]

**Automated pond library construction, detailed protocol**

**Set up of the Agilent Bravo with LT head for Post Shearing 3.0X Cleanup**

**Post Shearing 3.0X Automated Cleanup deck preparation**

**(Figure 3A displays deck layout)**

1| Wipe down Agilent Bravo deck with 70% Ethanol.

2| Add a box of 180μl Tips to deck positions 1, 2 and 3.

3| Aliquot 155 μl of Agencourt AMPure XP beads in each well of a 96 well Eppendorf twin.tec plate, then place plate of AMPure XP beads Buffer at deck position 4.

4| Aliquot 200 μl of 70% Ethanol in each well of a 96 well Eppendorf plate, then place plate of 70% Ethanol at deck position 5.

5| Aliquot 100 μl of 10 mM Tris-HC pH 8.0 in each well of a 96 well Eppendorf plate, the place plate of 10 mM Tris-HCl pH 8.0 at deck position 6.

6| Place the sample plate at deck position 8.

7| Place Dynal MPC-96S magnet plate at deck position 9.

8| Unscrew the bolt used to secure the plate pad to deck position 7, and the screw to attach the shearing rack holder/adapter to the plate pad and deck at deck position 7.

**Process Steps automated on the Bravo**

1| Put on 180 μl from tip box # 1.

2| Aspirate 50 μl of Agencourt AMPure XP beads from the Agencourt AMPure XP beads source plate, and dispense into sample plate.

3| Aspirate 65 μl of sheared sample from AFA tubes with in the shearing rack dispense into sample plate.

4| Aspirate 100 μl of Agencourt AMPure XP beads from the Agencourt AMPure XP beads source plate, and dispense into AFA shearing tubes located in the shearing rack at deck position 7.

5| Perform a Dual Height Mix to ensure the AMPure XP beads are properly mixed. NOTE: This step is used as a rinse to ensure all of the sample has been transferred from the shearing tubes to the sample plate.

6| Aspirate 100 μl of Agencourt AMPure XP beads from the AFA shearing tubes located at deck position 7 and dispense into sample plate.

7| Perform a Dual Height Mix to ensure the AMPure XP beads are properly mixed.

8| Allow the sample plate to sit for 5 minutes, after which time place the sample plate on a Dynal MPC – 96 S plate magnet for 8 minutes to allow the AMPure XP beads to separate from the solution.

9| Remove and discard the supernatant into the Agencourt AMPure XP beads source plate.

10| Discard the used 180 μl tips into tip box # 1.

11| Put on 180 μl tips from tip box # 2

12| Leaving the sample plate on the Dynal MPC-96S magnet plate, aspirate 60 μl of 70% EtOH from the 70% EtOH source plate and dispense into the sample plate. DO NOT MIX.

13| Allow the AMPure XP beads and sample sit in the 70% EtOH for 30 seconds, then remove the EtOH and discard into the 70% EtOH source plate.

14| Discard tips into 180 μl Tip Box # 2.

15| Move the sample plate off of the Dynal MPC-96s magnet plate and allow the sample-AMPure bead complex to air dry for approximately 6 minutes at room temperature.

16| Put on 180 μl from tip box # 3.

17| Aspirate 40μl of Tris-HCl pH 8.0 from the Tris-HCl pH 8.0 source plate, and dispense into sample plate.

18| Pipette mix 25x. Using ABI optical caps, seal Eppendorf plate containing samples.

19| Discard used 180 μl tips into tip box # 3.

20| Proceed to Automated/Manual End Repair Master Mix addition.


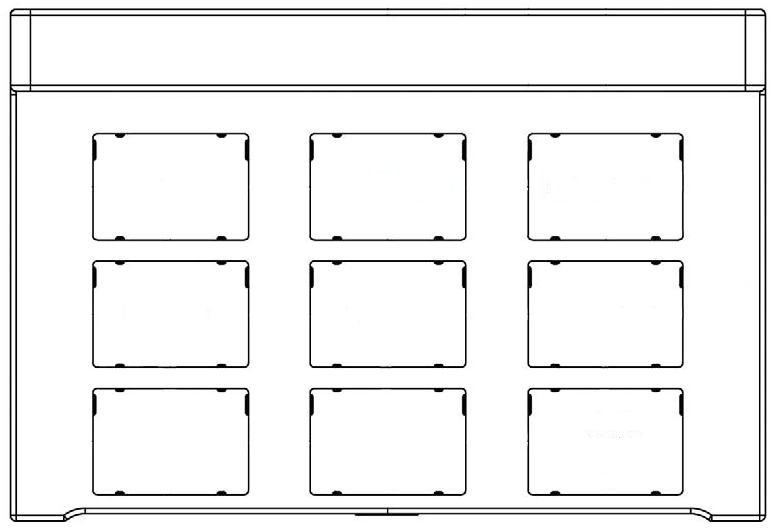


200ul Tips for binding and waste remove

200ul Tips for 70% Ethanol Wash

200ul Tips for elution and final transfer

96-well Eppendorf PCR plate with AMPure XP beads

96-well Eppendorf PCR plate with 70% Ethanol

96-well Eppendorf PCR plate with Elution Buffer

Shearing Tube Rack Adapter

Sample Plate

96-well PCR plate side magnet (Invitrogen)

**Set up of the Agilent Bravo with LT head for Post Shearing 3.0X Cleanup**

**Post Shearing 3.0X Automated Cleanup deck preparation**

**(Figure 3A displays deck layout)**

1| Wipe down Agilent Bravo deck with 70% Ethanol.

2| Add a box of 180μl tips to deck positions 1, 2 and 3.

3| Aliquot 155 μl of Agencourt AMPure XP beads in each well of a 96 well Eppendorf twin.tec plate, then place plate at deck position 4.

4| Aliquot 110 μl of 70% Ethanol in each well of a 96 well Eppendorf plate, then place plate at deck position 5.

5| Aliquot 50 μl of 10 mM Tris-HC pH 8.0 in each well of a 96 well Eppendorf plate, the place plate at deck position 6.

6| Place the sample plate at deck position 8.

7| Place Dynal MPC-96S magnet plate at deck position 9.

8| Unscrew the bolt used to secure the plate pad to deck position 7, and the use screw to attach the shearing rack holder/adapter to the plate pad and deck at deck position 7.

**Process Steps automated on the Bravo**

1| Put on 180 μl tips from tip box # 1.

2| Mix beads for initial aspiration. Aspirate 50 μl of Agencourt AMPure XP beads and dispense into sample plate.

3| Aspirate 65 μl of sheared sample from AFA tubes with in the shearing rack dispense into sample plate.

4| Aspirate 100 μl of Agencourt AMPure XP beads at deck position 4 and dispense into AFA shearing tubes located in the shearing rack at deck position 7.

6| Aspirate 100 μl of Agencourt AMPure XP beads from the AFA shearing tubes located at deck position 7 and dispense into sample plate at location 8.

7| Perform a 15 Dual Height Mix of 150ul to ensure the AMPure XP beads and sample are properly mixed.

8| Allow the sample plate to sit for 2 minutes, after which time place the sample plate on a Dynal MPC – 96 S plate magnet for 4 minutes. This will allow the AMPure XP beads to separate from the solution.

9| Remove and discard the supernatant into the Agencourt AMPure XP beads source plate.

10| Discard the used 180 μl tips into tip box # 1.

11| Put on 180 μl tips from tip box # 2.

12| Leaving the sample plate on the Dynal MPC-96S magnet plate, aspirate 100 μl of 70% EtOH and dispense into the sample plate. DO NOT MIX.

13| Allow the AMPure XP beads and sample to sit in the 70% EtOH for 30 seconds, then remove the EtOH and discard into the 70% EtOH source plate.

14| Discard tips into 180 μl Tip Box # 2.

11| Move the sample plate off of the Dynal MPC-96S magnet plate and allow the sample-AMPure bead complex to air dry for approximately 4 minutes at room temperature.

12| Put on 180 μl tips from tip box # 3.

13| Aspirate 40μl of Tris-HCl pH 8.0 and dispense into the sample plate.

14| Pipette mix 25x.

15| Discard used 180 μl tips into tip box # 3.

16| Using ABI optical caps, seal Eppendorf plate containing samples.

17| Proceed to Automated/Manual End Repair Master Mix addition.


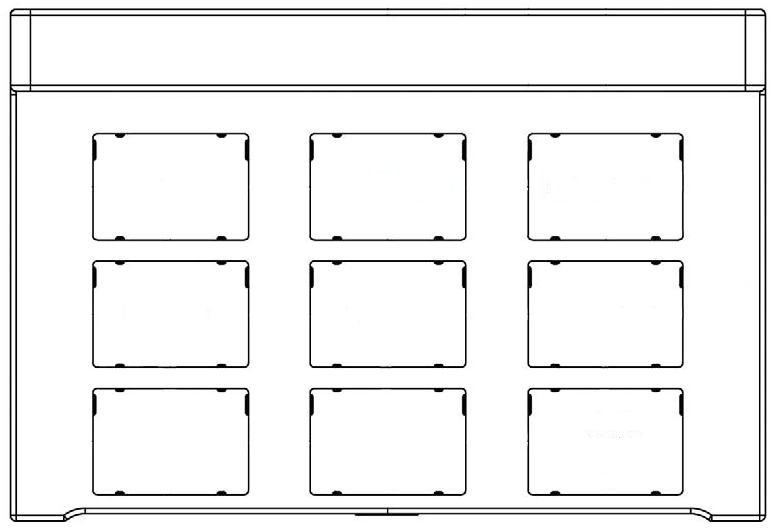


200ul Tips for binding and waste remove

200ul Tips for 70% Ethanol Wash

200ul Tips for elution and final transfer

96-well Eppendorf PCR plate with AMPure XP beads

96-well Eppendorf PCR plate with 70% Ethanol

96-well Eppendorf PCR plate with Elution Buffer

Shearing Tube Rack Adapter

Sample Plate

96-well PCR plate side magnet (Invitrogen)

**Set up of the Agilent Bravo with ST head for End Repair Mastermix addition**

1| Wipe down Agilent Bravo deck with 70% Ethanol.

2| Place a clean box of 70 ml 384ST V11 tips in position 6 and an empty 70 ml 384ST V11 tip box in position 3.

3| Place low volume insert holder in position 7 of the Bravo deck and place a clean 5 ml Deerac disposable reservoir in Column #3.

4| Open VWorks software and End Repair Mastermix dispense program.

**End Repair Mastermix Preparation**

1| Thaw the reagents on ice.

2| Once the reagents have thawed, prepare the appropriate amount of mastermix for the samples plus an additional 10 samples worth of reagent to account for dead volume as detailed in Table 1.

Table 1: Reagents Used for End repair Mastermix Preparation

3| Once the reagents have been combined, gently mix the mastermix, then place back on ice.

**Automated end Repair Addition Deck Preparation**

1| Place up to 5 ml of the prepared mastermix in the disposable 5ml Deerac reservoir located in column 3 of the low volume insert holder.

2| Select the appropriate script for the end repair mastermix dispense.

3| Start the script and monitor the dispense of the mastermix to ensure that large quantities of air are not being aspirated thereby reducing the amount of mastermix being dispensed (approximately 27 μl of End Repair mastermix per sample).

4| Once the protocol is complete, seal wells containing sample with ABI optical caps and place the sample plate on thermocycler. (Thermoprofile consist solely of an initial incubation of 12°C for 15 minutes followed by 12°C for 15 minutes then a held at 4°C indefinitely).

5| Once the incubation step has completed, proceed to End Repair Clean-up.


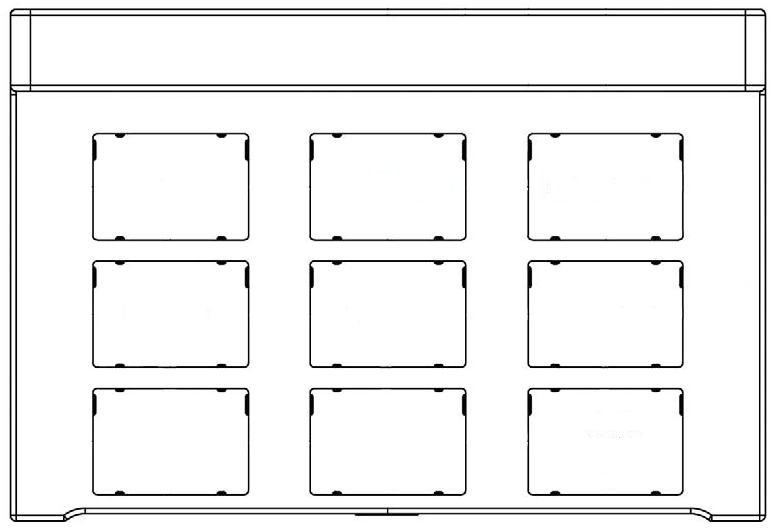


Empty 70 ul Tip Box for tip trash

70 ul Tips for mastermix dispense

Low volume insert holder

Sample plate

**End Repair Automated Mastermix Dispense Protocol**

1| Set head mode to 1 column: 12.

2| Pick up clean 70µl ST V11 Tips from quadrant 1 in column one from a clean 70 µl ST V11 Tips box located at position 3 on the Agilent Bravo deck. (Tips only need to be present in quadrants 1 and 2 of each position in which a sample is located).

3| Aspirate 27 µl of End Repair Mastermix from the 5ml Deerac disposable reservoir located in column 3 of the low volume insert holder located at position 7 on the Agilent Bravo deck.

4| Dispense the 27µl of End Repair Mastermix into samples located in Column one of the sample plate located at position 8 of the Agilent Bravo.

5| Tips are knocked off for disposal at quadrant 1 in column one of an empty 70 µl ST V11.

6| Repeat steps 1 through 3 for all subsequent columns on the sample which contain samples.
Clean tips should be used each time mastermix is aliquotted into a new column on the sample plate. For column 1, put the tips on in column 1 of the tip box and off in column 1 of the tip trash. For column 2 of the sample plate, put the tips on in column 3 of the tip box and off in column 3 of the tip trash. For column 3 of the sample plate, put the tips on in column 5 of the tip box and off in column 5 of the tip trash. And so on.

7| Pick up clean 70 µl ST V11 Tips from quadrant 2 of a clean 70 µl ST V11 Tips box located at position 3 on the Agilent Bravo deck.

8| Perform a Dual Height Mix on the wells containing sample and mastermix. Aspirate 40 μl at a height of 1 mm from the bottom of the well and dispensing 40 ml from the bottom of the well and dispensing at a height of 5 mm. Mix approximately 15 times.

9| Knock off tips for disposal into each quadrant 2 of an empty 70µl St V11.

**Set up of the Agilent Bravo with LT head for End Repair 2.2X Cleanup**

**End Repair 2.2X Automated Cleanup deck preparation**

**(Figure 3A displays deck layout)**

1| Wipe down Agilent Bravo deck with 70% Ethanol.

2| Add a box of 180μl tips to deck positions 1, 2 and 3.

3| Aliquot 150 μl of 20 % PEG 2.5M NaCl Buffer in each well of a 96 well Eppendorf twin.tec plate, then place plate at deck position 4.

4| Aliquot 110 μl of 70% Ethanol in each well of a 96 well Eppendorf plate, then place plate at deck position 5.

5| Aliquot 50 μl of 10 mM Tris-HC pH 8.0 in each well of a 96 well Eppendorf plate, then place plate at deck position 6.

6| Place the sample plate at deck position 8.

7| Place Dynal MPC 96S magnet plate at deck position 9.

**Process Steps automated on the Bravo**

1| Put on 180 μl tips from tip box # 1.

2| Aspirate 147.4 μl of 20% PEG 2.5M NaCl from the 20%PEG 2.5M NaCl source plate, and dispense into sample plate.

3| Perform a Dual Height Mix to ensure the AMPure XP beads are properly resuspended in 20% PEG 2.5 M NaCl buffer. Be sure to set the aspiration height to 1.5 mm from the bottom of the well and the dispense height to 13 mm from the bottom of the well. Mix approximately 130 μl 12 times.

4| Allow the sample plate to sit for 2 minutes, after which time place the sample plate, on the Dynal MPC – 96 S plate magnet for 4 minutes to allow the AMPure XP beads to separate from the solution.

5| Remove and discard the supernatant into the 20% PEG 2.5M NaCl source plate.

6| Discard the used 180 μl tips into tip box # 1.

7| Put on 180 μl tips from tip box # 2.

8| Leaving the sample plate on the Dynal MPC-96S magnet plate, aspirate 100 μl of 70% EtOH and dispense into the sample plate. DO NOT MIX.

9| Allow the AMPure XP beads and sample sit in the 70% EtOH for 30 seconds, then remove the EtOH and discard into the 70% EtOH source plate.

10| Discard tips into 180 μl Tip Box # 2.

11| Move the sample plate off of the Dynal MPC-96s magnet plate and allow the sample-AMPure bead complex to air dry for approximately 4 minutes at room temperature.

12| Put on 180 μl tips from tip box # 3.

13| Aspirate 40μl of Tris-HCl pH 8.0 and dispense into sample plate.

14| Perform a Dual height mix. Be sure to set the aspiration height to 1.5 mm from the bottom of the well and the dispense height to 6 mm from the bottom of the well. Mix approximately 40 μl 15 times.

15| Discard used 180 μl tips into tip box # 3.

16| Using ABI optical caps, seal Eppendorf plate containing samples.

17| Proceed to Automated/Manual A Base Master Mix addition.


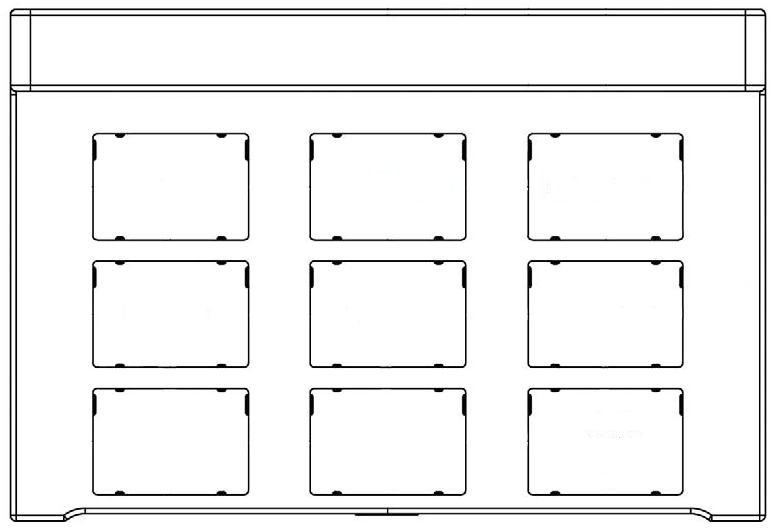


200μl Tips for binding and waste remove

200μl Tips for 70% Ethanol Wash

200μl Tips for elution and final transfer

96-well Eppendorf PCR plate with 20% PEG 2.5M NaCl

96-well Eppendorf PCR plate with 70% Ethanol

96-well Eppendorf PCR plate with Elution Buffer

Sample Plate

96-well PCR plate side magnet (Invitrogen)

**Set up of the Agilent Bravo with ST head for Automated A Base Addition Mastermix Addition**

1| Wipe down Agilent Bravo deck with 70% Ethanol.

2| Place a clean box of 70 ml l 384ST V11 tips in position 6 and an empty 70 ml 384ST V11 tip box in position 3.

3| Place low volume insert holder in position 7 of the Bravo deck and place a clean 5 ml Deerac disposable reservoir in Column #3.

4| Open VWorks software.

**A Base Addition Mastermix Preparation**

1| Thaw the reagents on ice.

2| Once the reagents have thawed, prepare the appropriate amount of mastermix for the samples plus an additional 10 samples to account for dead volume as detailed in Table 2.

Table 2: Reagents Used for A Base Mastermix Preparation

3| Once the reagents have been combined, gently mix the mastermix, then place back on ice.

**Automated A Base Addition Deck Preparation**

1| Place up to 5 ml of the prepared A Base Addition mastermix in the disposable 5ml Deerac reservoir located in column 3 of the low volume insert holder.

2| Select the appropriate script for the A Base Addition mastermix dispense.

3| Start the script and monitor the dispense of the mastermix to ensure that large quantities of air are not being aspirated thereby reducing the amount of mastermix being dispensed (approximately 20 μl of A Base Addition master mix per sample).

4| Once the protocol is complete, seal well containing sample with ABI optical caps and place the sample plate on thermocycler. (Thermoprofile consists solely of 37°C for 30 minutes then held at 4°C indefinitely).

5| Once the incubation step has completed, proceed to A Base Addition Clean-up.


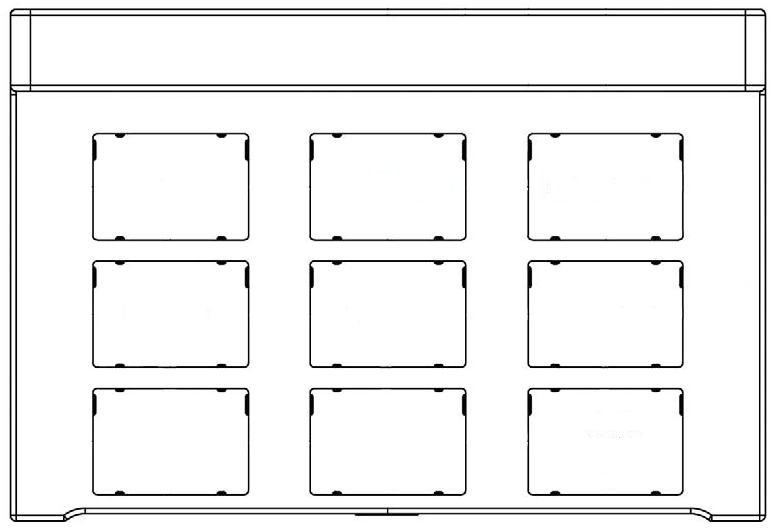


Empty 70 ul Tip Box for tip trash

70 ul Tips for mastermix dispense

Low volume insert holder

Sample plate

**Automated A Base Addition Mastermix Dispense Protocol**

1| Set head mode to 1 column:12.

2| Pick up clean 70µl ST V11 Tips from quadrant 1 in column one from a clean 70 µl ST V11 Tips box located at position 3 on the Agilent Bravo deck. (Tips only need to be present in quadrants 1 and 2 of each position in which a sample is located).

3| Aspirate 20 µl of A Base Addition Mastermix from the 5ml Deerac disposable reservoir located in column 3 of the low volume insert holder located at position 7 on the Agilent Bravo deck.

4| Dispense the 20 µl of A Base Addition Mastermix into samples located in Column one of the sample plate located at position 8 of the Agilent Bravo.

5| Tips are knocked off for disposal into each quadrant 1 in column one of an empty 70 µl St V11.

6| Repeat steps 1 through 3 for all subsequent columns on the sample which contain samples.
Clean tips should be used each time mastermix is aliquotted into a new column on the sample plate. For column 1, put the tips on in column 1 of the tip box and off in column 1 of the tip trash. For column 2 of the sample plate, put the tips on in column 3 of the tip box and off in column 3 of the tip trash. For column 3 of the sample plate, put the tips on in column 5 of the tip box and off in column 5 of the tip trash. And so on.

7| Pick up clean 70 µl ST V11 Tips from quadrant 2 of a clean 70 µl ST V11 Tips box located at position 3 on the Agilent Bravo deck.

8| Perform a Dual Height Mix on the wells containing sample and mastermix. Aspirate 40 μl at a height of 1 mm from the bottom of the well and dispensing 40 ml from the bottom of the well and dispensing at a height of 5 mm. Mix approximately 15 times.

9| Knock off tips for disposal into quadrant 2 of an empty 70 µl St V11.

**Set up of the Agilent Bravo with LT head for A Base Addition 2.2X Cleanup**

**A Base Addition 2.2X Automated Cleanup deck preparation**

**(Figure 3A displays deck layout)**

1| Wipe down Agilent Bravo deck with 70% Ethanol.

2| Add a box of 180μl tips to deck positions 1, 2 and 3.

3| Aliquot 150 μl of 20 % PEG 2.5M NaCl Buffer in each well of a 96 well Eppendorf twin.tec plate, then place plate at deck position 4.

4| Aliquot 110 μl of 70% Ethanol in each well of a 96 well Eppendorf plate, then place plate at deck position 5.

5| Aliquot 50 μl of 10 mM Tris-HC pH 8.0 in each well of a 96 well Eppendorf plate, then place plate at deck position 6.

6| Place the sample plate at deck position 8.

7| Place Dynal MPC 96S magnet plate at deck position 9.

**Process Steps automated on the Bravo**

1| Put on 180 μl tips from tip box # 1.

2| Aspirate 132 μl of 20% PEG 2.5M NaCl and dispense into sample plate.

3| Perform a Dual Height Mix to ensure the AMPure XP beads are properly resuspended in 20% 2.5 M NaCl buffer. Be sure to set the aspiration height to 1.5 mm from the bottom of the well and the dispense height to 13 mm from the bottom of the well. Mix approximately 130 μl 15 times.

4| Allow the sample plate to sit for 2 minutes, after which time place the sample plate, on the Dynal MPC – 96S plate magnet for 4 minutes to allow the AMPure XP beads to separate from the solution.

5| Remove and discard the supernatant into the 20% PEG 2.5M NaCl source plate

6| Discard the used 180 μl tips into tip box # 1.

7| Put on 180 μl tips from tip box # 2.

8| Leaving the sample plate on the Dynal MPC-96S magnet plate, aspirate 100 μl of 70% EtOH and dispense into the sample plate. DO NOT MIX.

9| Allow the AMPure XP beads and sample sit in the 70% EtOH for 30 seconds, then remove the EtOH and discard into the 70% EtOH source plate.

10| Discard tips into 180 μl Tip Box # 2.

11| Move the sample plate off of the Dynal MPC-96S magnet plate and allow the sample-AMPure bead complex to air dry for approximately 4 minutes at room temperature

12| Put on 180 μl tips from tip box # 3.

13| Aspirate 40μl of Tris-HCl pH 8.0 and dispense into sample plate.

14| Perform a Dual height mix . Be sure to set the aspiration height to 1.5 mm from the bottom of the well and the dispense height to 6 mm from the bottom of the well. Mix approximately 40 μl 15 times.

15| Discard used 180 μl tips into tip box # 3.

16| Using ABI optical caps, seal Eppendorf plate containing samples.

17| Proceed to Automated/Manual Adapter Ligation Master Mix addition.


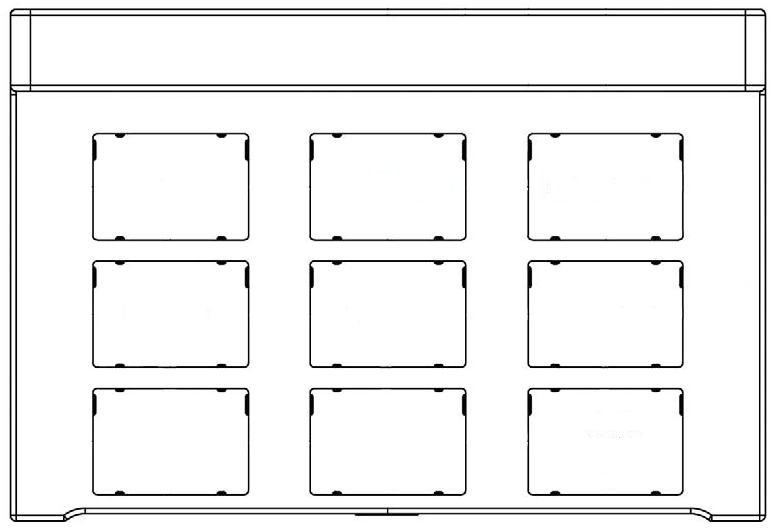


200ul Tips for binding and waste remove

200ul Tips for 70% Ethanol Wash

200ul Tips for elution and final transfer

96-well Eppendorf PCR plate with 20% PEG 2.5M NaCl

96-well Eppendorf PCR plate with 70% Ethanol

96-well Eppendorf PCR plate with Elution Buffer

Sample Plate

96-well PCR plate side magnet (Invitrogen)

**Set up of the Agilent Bravo with ST head for Automated Adapter Ligation Mastermix Addition**

1| Wipe down Agilent Bravo deck with 70% Ethanol.

2| Place a clean box of 70 ml 384ST V11 tips in position 6 and an empty 70μl 384ST V11 tip box in position 3.

3| Place low volume insert holder in position 7 of the Bravo deck and place a clean 5 ml Deerac disposable reservoir in Column #3.

4| Open VWorks software

**Adapter Ligation Mastermix Preparation**

1| Thaw reagents on ice. Thaw enough for the number of samples being run plus an extra 15 samples for to account for dead volume.

2| Prepare Adapter Ligation Mastermix as described in Table 1.

Table 1: Reagents used for Adapter Ligation mastermix preparation

3| Thaw the matrix rack containing 0.5ml matrix 2-D barcoded tubes with at least 5 μl of adapters in each tube at room temperature. Each sample should have its own tube of adapter aliquot, and said tube should be placed in the same location in the matrix rack as the sample is within the sample plate.

4| Once thawed vortex the 2-D matrix tubes containing the adapters at a moderate speed followed by a quick spin down.

5| Decap the 2-D matrix tubes containing the adapters, and place the matrix rack on position 9 of the Agilent Bravo.

6| Place up to 5 ml of the prepared mastermix in the disposable 5ml Deerac reservoir located in column 3 of the low volume insert holder.

7| Select the appropriate script for the Adapter Ligation mastermix dispense.

8| Start the script and monitor the dispense of the mastermix to ensure that large quantities of air are not being aspirated thereby reducing the amount of mastermix being dispensed (approximately 15 μl of Adapter Ligation mastermix per sample and 3 μl of paired end adpater).

9| Once the protocol is complete, seal wells containing sample with ABI optical caps and place the sample plate on thermocycler. (Thermoprofile consists solely of 25°C for 15 minutes then held at 4°C indefinitely).

10| Once the incubation step has completed, remove the sample plate from the thermocycler and proceed to Adapter Ligation Clean-up.


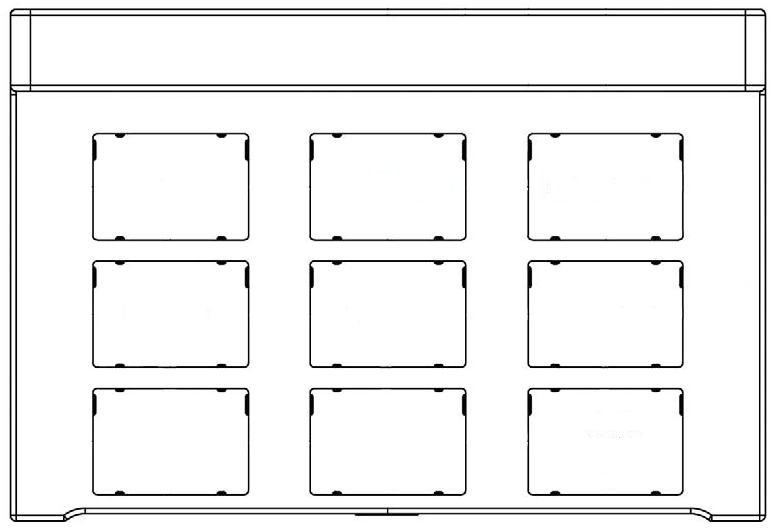


Empty 70 ul Tip Box for tip trash

70 ul Tips for mastermix dispense

Low volume insert holder

Sample plate

0,5ml Matrix tubes with PE Adapters

**Automated Adapter Ligation Mastermix Dispense Protocol**

1| Set head mode to 1 column:12.

2| Pick up clean 70 µl ST V11 Tips from position 1 of each quadrant in column one from a clean 70 µl ST V11 Tips box located at position 3 on the Agilent Bravo deck. (Tips only need to be present in quadrants 1 and 2 of each position in which a sample is located)

3| Aspirate 15 µl of Adapter Ligation Mastermix from the 5ml Deerac disposable reservoir located in column 3 of the low volume insert holder located at position 7 on the Agilent Bravo deck.

4| Dispense the 15 µl of Adapter Ligation Mastermix into samples located in Column one of the sample plate located at position 8 of the Agilent Bravo.

5| Knock off tips for disposal into quadrant 1 in column one of an empty 70 µl St V11.

6| Repeat steps 1 through 3 for all subsequent columns on the sample which contain samples.
Clean tips should be used each time mastermix is aliquotted into a new column on the sample plate. For column 1, put the tips on in column 1 of the tip box and off in column 1 of the tip trash. For column 2 of the sample plate, put the tips on in column 3 of the tip box and off in column 3 of the tip trash. For column 3 of the sample plate, put the tips on in column 5 of the tip box and off in column 5 of the tip trash. And so on.

7| Pick up clean 70 µl ST V11 Tips from quadrant 2 of a clean 70 µl ST V11 Tips box located at position 3 on the Agilent Bravo deck.

8| Aspirate 3 μl of adapter from 0.5ml 2-D barcoded matrix tubes contained within the 0.5ml Matrix rack located at position 9 on the Agilent Bravo.

9| Dispense 3 μl of adapter into the corresponding wells of the sample plate. NOTE: Do not discard tips. They will be used to mix sample.

10| Perform a Dual Height Mix on the wells containing sample and mastermix. Aspirate 40 μl at a height of 1 mm from the bottom of the well and dispensing 40 ul from the bottom of the well and dispensing at a height of 5 mm. Mix approximately 15 times.

11| Knock off tips for disposal into quadrant 2 of an empty 70 µl St V11.

**Set up of the Agilent Bravo with LT head for Adapter Ligation 0.7X Cleanup**

**Adapter Ligation 0.7X Automated Cleanup deck preparation**

**(Figure 3A displays deck layout)**

1| Wipe down Agilent Bravo deck with 70% Ethanol.

2| Add a box of 180μl Tips to deck positions 1, 2 and 3.

3| Aliquot 50 μl of 20 % PEG 2.5M NaCl Buffer in each well of a 96 well Eppendorf twin.tec plate, then place the plate at deck position 4.

4| Aliquot 110 μl of 70% Ethanol in each well of a 96 well Eppendorf plate, then place the plate at deck position 5.

5| Aliquot 50 μl of 10 mM Tris-HC pH 8.0 in each well of a 96 well Eppendorf plate, then place the plate at deck position 6.

6| Place the sample plate at deck position 8.

7| Place an unused Eppendorf 96 well twin.tec plate at position 7 and mark as adapter ligated library.

8| Place Dynal MPC-96S magnet plate at deck position 9.

**Process Steps automated on the Bravo**

1| Put on 180 μl tips from tip box # 1.

2| Aspirate 40.6 μl of 20% PEG 2.5M NaCl and dispense into sample plate.

3| Perform a Dual Height Mix to ensure the AMPure XP beads are properly resuspended in the 20% PEG 2.5 M NaCl buffer. Be sure to set the aspiration height to 1.5 mm from the bottom of the well and the dispense height to 7 mm from the bottom of the well. Mix approximately 80 μl 15 times.

4| Allow the sample plate to sit for 2 minutes, after which time place the sample plate on a Dynal MPC – 96 S plate magnet for 4 minutes to allow the AMPure XP beads to separate from the solution.

5| Remove and discard the supernatant into the 20% PEG NaCl 2.5M source plate.

6| Discard the used 180 μl tips into tip box # 1.

7| Put on 180 μl tips from tip box # 2

8| Leaving the sample plate on the Dynal MPC-96S magnet plate, aspirate 100 μl of 70% EtOH and dispense into the sample plate. DO NOT MIX.

9| Allow the AMPure XP beads and sample sit in the 70% EtOH for 30 seconds, then remove the EtOH and discard into the 70% EtOH source plate.

10| Discard tips into 180 μl Tip Box # 2.

11| Move the sample plate off of the Dynal MPC-96S magnet plate and allow the sample-AMPure bead complex to air dry for approximately 4 minutes at room temperature.

12| Put on 180 μl tips from tip box # 3.

13| Aspirate 40μl of Tris-HCl pH 8.0 and dispense into sample plate.

14 Perform a Dual height mix. Be sure to set the aspiration height to 1.5 mm from the bottom of the well and the dispense height to 6 mm from the bottom of the well. Mix approximately 40 μl 15 times.

15| Allow the resuspended sample to sit for approximately 2 minutes.

16| Place the sample plate, on a Dynal MPC – 96S plate magnet for 3 minutes to allow the AMPure XP beads to separate from the solution.

17| Aspirate the eluate and dispense into the Eppendorf 96 well twin.tec plate located at position 7.

18| Discard tips into tip box # 3.

19| Using ABI optical caps, seal Eppendorf plate containing samples.

20| Proceed to Automated/Manual Pond Enrichment Master Mix addition.


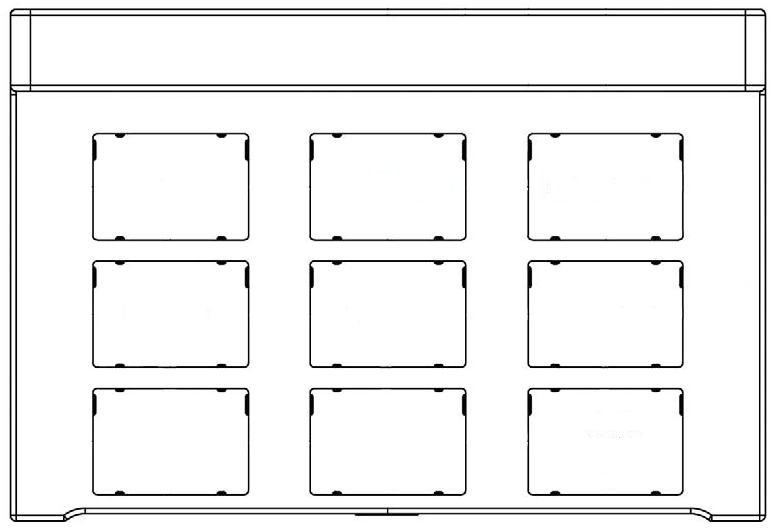


200ul Tips for binding and waste remove

200ul Tips for 70% Ethanol Wash

200ul Tips for elution and final transfer

96-well Eppendorf PCR plate with 20% PEG 2.5 M NaCl

96-well Eppendorf PCR plate with 70% Ethanol

96-well Eppendorf PCR plate with Elution Buffer

96-well Eppendorf PCR plate for final elute transfer

Sample Plate

96-well PCR plate side magnet (Invitrogen)

**Set up of the Agilent Bravo with ST head for Pond Enrichment addition**

1| Wipe down Agilent Bravo deck with 70% Ethanol.

2| Place a clean box of 70 ul 384ST V11 tips in position 6 and an empty 70 ul 384ST V11 tip box in position 3.

3| Place low volume insert holder in position 7 of the Bravo deck and place a clean 5 ml Deerac disposable reservoir in Column #3.

4| Open VWorks software.

**Pond Enrichment Mastermix Preparation**

1| Thaw the reagents on ice.

2| When the reagents have thawed, prepare the appropriate amount of mastermix for the samples plus and additional 10 samples to account for dead volume as detailed in Table 1.

Table 1: Reagents Used for Pond Enrichment Mastermix Preparation

3| Once the reagents have been combined, gently mix the mastermix, then place back on ice.

**Automated Pond Enrichment Addition Deck Preparatio**n

1| Place up to 5 ml of the prepared Pond Enrichment mastermix in the disposable 5ml Deerac reservoir located in column 3 of the low volume insert holder.

2| Select the appropriate script for the Pond Enrichment mastermix dispense.

3| Start the script and monitor the dispense of the mastermix to ensure that large quantities of air are not being aspirated thereby reducing the amount of mastermix being dispensed (approximately 20 μl of Pond Enrichment mastermix per sample).

4| Once the protocol is complete, seal well containing sample with ABI optical caps and place the sample plate on thermocycler. (Thermoprofile detailed in Table 2).

5| Once the incubation step has completed, proceed to Pond Enrichment Clean-up.

Table 2: Pond Enrichment Thermoprofile


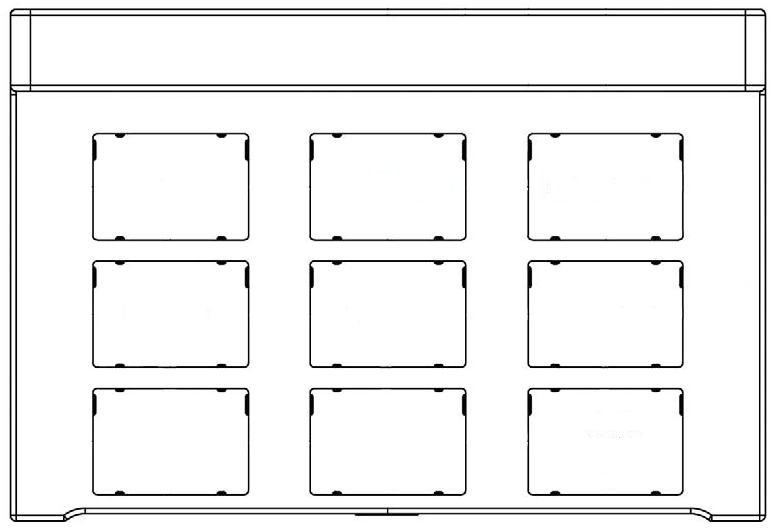


Empty 70 ul Tip Box for tip trash

70 ul Tips for mastermix dispense

Low volume insert holder

Sample plate

Pond Enrichment Automated Mastermix Dispense Protocol

1| Set head mode to 1 column: 12.

2| Pick up clean 70 µl ST V11 Tips from quadrant 1 in column one from a clean 70 µl ST V11 Tips box located at position 3 on the Agilent Bravo deck. (Tips only need to be present in quadrants 1 and 2 of each position in which a sample is located).

3| Aspirate 20 µl of Pond Enrichment Mastermix from the 5ml Deerac disposable reservoir located in column 3 of the low volume insert holder located at position 7 on the Agilent Bravo deck.

4| Dispense the 20 µl of Pond Enrichment Mastermix into samples located in Column one of the sample plate located at position 8 of the Agilent Bravo.

5| Knock off tips for disposal into quadrant 1 in column one of an empty 70 µl St V11.

6| Repeat steps 1 through 3 for all subsequent columns on the sample which contain samples.
Clean tips should be used each time mastermix is aliquotted into a new column on the sample plate. For column 1, put the tips on in column 1 of the tip box and off in column 1 of the tip trash. For column 2 of the sample plate, put the tips on in column 3 of the tip box and off in column 3 of the tip trash. For column 3 of the sample plate, put the tips on in column 5 of the tip box and off in column 5 of the tip trash. And so on.

7| Pick up clean 70 µl ST V11 Tips from quadrant 2 of a clean 70 µl ST V11 Tips box located at position 3 on the Agilent Bravo deck.

8| Perform a Dual Height Mix on the wells containing sample and mastermix. Aspirate 40 μl at a height of 1 mm from the bottom of the well and dispensing 40 ml from the bottom of the well and dispensing at a height of 5 mm. Mix approximately 15 times.

9| Knock off tips for disposal into quadrant 2 of an empty 70 µl St V11.

**Set up of the Agilent Bravo with LT head for Pond Enrichment 1.8 X Cleanup**

**Pond Enrichment 1.8X Automated Cleanup deck preparation**

**(Figure 3A displays deck layout)**

1| Wipe down Agilent Bravo deck with 70% Ethanol.

2| Add a box of 180μl Tips to deck positions 1, 2 and 3.

3| Aliquot 120 μl of Agencourt AMPure XP beads in each well of a 96 well Eppendorf twin.tec plate, then place the plate at deck position 4.

4| Aliquot 110 μl of 70% Ethanol in each well of a 96 well Eppendorf plate, then place the plate at deck position 5.

5| Aliquot 50 μl of 10 mM Tris-HC pH 8.0 in each well of a 96 well Eppendorf plate, then place the plate at deck position 6.

6| Place the sample plate at deck position 8.

7| Place an unused Eppendorf 96 well twin.tec plate at position 7 and mark as enriched library.

8| Place Dynal MPC 96-S magnet plate at deck position 9.

**Process Steps automated on the Bravo**

1| Put on 180 μl tips from tip box # 1. ``

2| Aspirate 108 μl of Agencourt AMPure XP beads and dispense into sample plate.
Mix beads prior to aspiration

3| Perform a Dual Height Mix (mixing approximately 15 times), then allow the sample plate to sit for approximately 2 minutes. Be sure to set the aspiration height to 1.5 mm from the bottom of the well and the dispense height to 7 mm from the bottom of the well. Mix approximately 155 μl 15 times.

4| Place the sample plate on a Dynal MPC – 96 S plate magnet for 4 minutes to allow the AMPure XP beads to separate from the solution.

5| Remove and discard the supernatant into the AMpure XP beads source plate.

6| Discard the used 180 μl tips into tip box # 1.

7| Put on 180 μl tips from tip box # 2.

8| Leaving the sample plate on the Dynal MPC-96S magnet plate, aspirate 100 μl of 70% EtOH and dispense into the sample plate. DO NOT MIX.

9| Allow the AMPure XP beads and sample sit in the 70% EtOH for 30 seconds, then remove the EtOH and discard into the 70% EtOH source plate.

10| Discard tips into 180 μl Tip Box # 2.

11| Move the sample plate off of the Dynal MPC-96S magnet plate and allow the sample-AMPure bead complex to air dry for approximately 4 minutes at room temperature

12| Put on 180 μl tips from tip box # 3.

13| Aspirate 40 ml of Tris-HCl pH 8.0 and dispense into sample plate.

14| Perform a Dual height mix. Be sure to set the aspiration height to 1.5 mm from the bottom of the well and the dispense height to 6 mm from the bottom of the well. Mix approximately 40 μl 10 times.

15| Allow the resuspended sample to sit for approximately 2 minutes.

16| Place the sample plate, on a Dynal MPC–96S plate magnet for 3 minutes to allow the AMPure XP beads to separate from the solution.

17| Aspirate the eluate and dispense into the Eppendorf 96 well twin.tec plate located at position 7.

18| Discard tips into tip box # 3.

19| Using ABI optical caps, seal Eppendorf plate containing samples.

19| Proceed to sample quantification. If needed, normalize sample to 25 ng/μl using Tris-HCl pH 8.0 to dilute your sample.


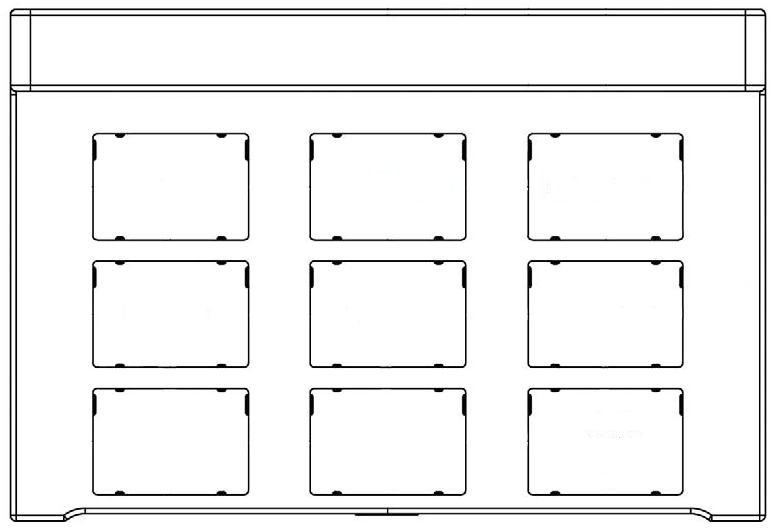


200ul Tips for binding and waste remove

200ul Tips for 70% Ethanol Wash

200ul Tips for elution and final transfer

96-well Eppendorf PCR plate with Ampure Beads

96-well Eppendorf PCR plate with 70% Ethanol

96-well Eppendorf PCR plate with Elution Buffer

96-well Eppendorf PCR plate for final elute transfer

Sample Plate

96-well PCR plate side magnet (Invitrogen)
