# Supplementary material for: A scalable, fully automated process for construction of sequence-ready human exome targeted capture libraries
Source: Genome Biol. 2011 Jan 4;12(1):R1. doi: 10.1186/gb-2011-12-1-r1 (PMC3091298; doi:10.1186/gb-2011-12-1-r1)
Supplement: Additional file 15 — Automated SHS hybridization and capture protocol. A Word document detailing the automated hybridization and capture protocols. [file gb-2011-12-1-r1-S15.DOCX]

**Automated hybridization and capture, detailed protocols**

**Automated Hybridization Set-up**

**Note: It is important to follow the steps of this protocol exactly as timing of the process is critical to performance of the selections.**

1| Fill two ice buckets with ice.

2| Thaw the following reagents on ice: 50X Denhardt’s Solution, Human Cot-1, Salmon Sperm, and SureSelect Blocking Agent # 3.

3| While the reagents are thawing, prepare heat block by heating it up to 65°C.

4| In a second ice bucket, place the bait aliquots and allow to thaw. Also, if your Ponds have been frozen for storage, thaw on ice.

5| Once the 50 X Denhardt’s solution is completely thawed begin to prepare the Hybridization buffer, as described in Table 1. Once the preparation of the hybridization buffer is complete, place the hybridization buffer on the 65°C heat block and allow the buffer to incubate for at least 15 minutes.

Table 1: Reagents Used for Hybridization buffer Preparation

6| While the hybridization buffer is incubating, prepare a 1:10 dilution (with nuclease free water) of Salmon sperm DNA for a final volume of 287.5μl.

7| Prepare the carrier DNA-Blocking Agent mixture by adding 287.5 μl of Human Cot-1 and 345 μl of SureSelect Block # 3 to the diluted Salmon Sperm. Lightly vortex to mix, then spin down.

8| Aliquot 76 μl of the carrier DNA-blocking agent mixture into 12 individual 0.5ml 2-D Matrix tubes and place the 0.5 ml tubes in row A of an empty 0.5 ml Matrix tube rack. Place tube rack on ice.

9| Once bait aliquots are completely thawed, add the appropriate amount of Superase and Control SNP fingerprinting bait. For every 5 ul of bait (100ng/ul), add 1 ul of Superase and 1 ul of Control SNP fingerprinting bait (360pg).

10| Gently pipette mix the baits then transfer the appropriate amount of Bait-Superase mixture into 0.5 ml tube. Place the 0.5 ml tube back on ice.

11| Prepare 2 of the 3 thermocyclers needed, by setting the block temperature to hold at 65°C and the lid to hold at 105°C.

**Hybridization Deck Set-up**

1| Wipe down Agilent Bravo deck with 70% ethanol.

2| Place a fresh box of 70 μl 384ST V11 tips in position 5 and an empty 70 μl 384ST V11 tip box in position 1.

3| Place Low volume insert holder in position 9 of the Bravo deck and place a fresh 5 ml Deerac disposable reservoir in Column # 1.

4| Label an Eppendorf 96-well twin.tec plate as Bait Plate, then place at deck position 3.

5| Label an Eppendorf 96-well twin.tec plate as Hybridization buffer Plate, then place at deck position 8.

6| Label an Eppendorf 96-well twin.tec plate as Target Plate, then place at deck position 7.

7| Decap the 0.5 ml Matrix tubes containing Bait-Superase mixture and place in row A of a 0.5 ml Matrix tube rack. Place the 0.5 ml Matrix tube rack at deck position 2.

8| Decap the 0.5 ml Matrix tubes containing Blocking mixture and place in row A of a 0.5 ml Matrix tube rack. Place the 0.5 ml Matrix tube rack at deck position 6.

9| Decap the 0.5 ml Matrix tubes containing DNA/Pond Source. Place the 0.5 ml Matrix tube rack at deck position 6.

10| Place up to 5 ml of the prepared warmed hybridization buffer (at 65°C) in the disposable 5ml Deerac reservoir located in column 1 of the Low volume insert holder.


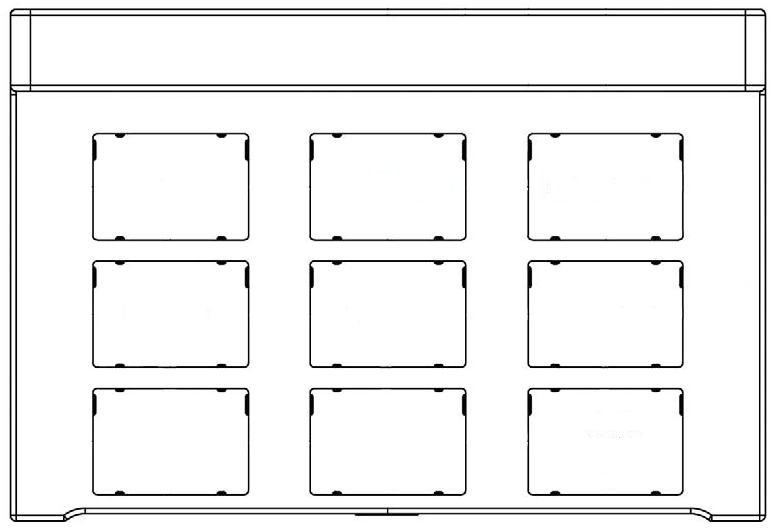


Tip Trash (70 ul Tip Box)

Bait Source (0.5 ml Matrix Tube Rack

Bait Plate (Eppendorf 96 well twin.tec)

DNA/Pond Source (0.5 ml Matrix Tube Rack

70 ul Tip Box

(384 ST70)

Blocking Agent Source (0.5 ml Matrix Tube Rack

Target Plate (Eppendorf 96 well twin.tec)

Hyb Buffer Plate (Eppendorf 96 well twin.tec

Low Insert Volume Holder

**Automated Hybridization Set-up**

Order of Automation step:

1. Bait dispense
2. Hybridization buffer dispense
3. Target plate preparation

Bait Plate Set-up

1| Set head mode to 1 row: 1

*NOTE: 70 µl tips should only be placed on row A of the ST head.*

2| Pick up fresh 70 µl ST V11 Tips from a fresh 70 µl ST V11 Tips box located at position 5 on the Agilent Bravo deck.

*NOTE: Be sure well selection is set to P1.*

3| Aspirate 58 µl of Bait-Superase mixture from 0.5 ml Matrix tube rack containing Bait source tubes (0.5 ml Matrix tubes) located at deck position 2 on the Agilent Bravo deck.

*NOTE: Be sure well selection is set to 1 selection: row A.*

4| Dispense the 7 µl of Bait-Superase mixture into Bait Plate located at position 3 of the Agilent Bravo.

*NOTE: Be sure well selection is set to 8 selections: rows A-H.*

5| Empty the remaining contents of the tips into the Bait-Superase source tubes.

6| Knock off tips for disposal at quadrant 1 of row A of an empty 70µl ST V11 located at deck position 1

*NOTE: Be sure well selection is set to 1 selection: rows A1.*

7| Pause protocol, remove the Bait Plate and seal well containing Bait-Superase mixture with ABI optical caps. Place the plate on ice.

Hybridization buffer Dispense

1| Set head mode to 1 column: 12

*NOTE: 70 µl tips should only be placed on column 1 of the ST head.*

2| Insert a Java Script (BuiltIn) immediately following the Set Head Mode in the protocol Script.

a) Java Script should be written as follows:

var column

loop=loop/8

loop=Math.ceil(loop)

3| Pick up fresh 70 µl ST V11 Tips from a fresh 70 µl ST V11 Tips box located at position 5 on the Agilent Bravo deck.

*NOTE: Be sure well selection to 1 selection: A1.*

4| Aspirate 5 µl from Hybridization buffer source

5| Open loop which will be run 12 times.

6| Within the loop, aspirate 40 µl of hybridization buffer from the Hybridization buffer source reservoir.

*NOTE: Be sure well selection to 1 selection: column 1.*

7| Following the hybridization buffer aspiration, dispense 40 µl of hybridization buffer into the Hybridization buffer Plate located at deck position 8.

*NOTE: Be sure well selection to 1 selection: column 12.*

8| Close loop.

9| Empty the remaining contents of the tips into the Hybridization buffer source reservoir.

*NOTE: Be sure well selection to 1 selection: column 1.*

10| Knock off tips for in an empty 70µl ST V11 located at deck position 1

*NOTE: Be sure well selection is set to 1 selection: rows A24.*

11| Pause protocol, remove the Hybridization Plate and seal well containing hybridization buffer mixture with ABI optical caps. Place the plate on one of the thermalcyclers that has been preheated to 65°C

Target Plate Preparation

1. *Blocking Agent Dispense*

1| Set head mode to 1 row: 8

2| Pick up fresh 70 µl ST V11 Tips from a fresh 70 µl ST V11 Tips box located at position 5 on the Agilent Bravo deck.

*NOTE: Be sure well selection is set to A2.*

3| Aspirate 66 µl of Blocking Agent mixture from 0.5 ml Matrix tube rack containing Blocking Agent source tubes (0.5 ml Matrix tubes) located at deck position 6 on the Agilent Bravo deck.

*NOTE: Be sure well selection is set to 1 selection: row A.*

4| Dispense the 8 µl of Blocking Agent mixture into the Target plate located at deck position 7 of the Agilent Bravo.

*NOTE: Be sure well selection is set to 8 selections: rows A-H.*

5| Empty the remaining contents of the tips into the Blocking Agent source tubes.

6| Knock off tips for disposal at quadrant 1 of row A column one of an empty 70µl ST V11 located at deck position 1.

*NOTE: Be sure well selection is set to 1 selection: rows P1.*

1. *DNA/Pond Dispense*

1| Pick up fresh 70 µl ST V11 Tips from a fresh 70 µl ST V11 Tips box located at position 5 on the Agilent Bravo deck.

*NOTE: Be sure well selection is set to quadrant 4.*

2| Aspirate 20 µl of DNA/Pond from 0.5 ml Matrix tube rack containing DNA/Pond source tubes (0.5 ml Matrix tubes) located at deck position 4 on the Agilent Bravo deck.

*NOTE: Be sure well selection is set to entire plate.*

4| Dispense the 20 µl of DNA/Pond into Target plate located at position 7 of the Agilent Bravo.

5| After sample has been dispensed in the Target plate, perform a dual height mix aspirating at a height of 1 mm and dispensing at a height of 1 mm. Mix a total of 5 times.

6| Knock off tips for disposal at quadrant 4 of row A column one of an empty 70µl ST V11 located at deck position 1.

7| Seal wells containing DNA-Blocking Agent mixture with ABI optical caps.

Hybridization

1| Prepare the third (non-incubating) thermocycler, by ensuring the following thermoprofile has been entered: 95°C for 5 minutes, the hold at 65°C. Name the cycling protocol Hybridization.

2| Place the target plate of the third (non-incubating) thermocycler, and start the hybridization protocol.

3| Monitor the time of the hybridization protocol on the thermocycler. When the time remaining reaches approximately 2 minutes for the 95°C denature step, place the bait plate on the second thermocycler incubating at 65°C. Be sure to close the lid of the thermalcycler once the Bait plate has been positioned securely.

4| Again monitor the time remaining on the hybridization protocol. When the time remaining reaches approximately 2 seconds for the 95°C denature step, pause the protocol and open the lid of the thermalcycler and remove the Optical Caps.

5| Immediately open the lids of the thermalcyclers containing the hybridization buffer plate and the bait plate and quickly remove the Optical Caps.

6| Quickly but carefully transfer 7 μl of the Bait-Superase mixture from the bait plate into the target Plate. Pipette mix 3 times. NOTE: Be sure the Superase-Bait mixture is being added to well of the target plate that contain sample and hybridization buffer.

7| Quickly but carefully transfer 35 μl of the hybridization buffer from the hybridization buffer plate into the target Plate. Pipette mix 3 times. NOTE: Be sure the hybridization buffer is being added to well of the target plate that contains sample.

8| Once the Bait-Superase mixture and the hybridization buffer has been added to the target plate, pipette mix all wells containing sample, approximately 10 times to ensure the mixture is homogenous.

9| Seal the wells of the Target plate using caps, close the lid of the thermalcycler and resume the hybridization cycling program.

10| Allow the hybridization program to continue running for approximately 21 to 70 hours.

11| After the Target plate has run on the hybridization program for the desired length of time, remove the Target plate and proceed to the Capture protocol.

**Automated Capture Protocol deck preparation**

**Set up of the Agilent Bravo with LT head for Automated Capture Protocol**

(Figure below displays deck layout)

Manual Process Steps

1| BW Buffer

A) 40 ml nuclease-free water

B) 10 ml 5M NaCl

C) 50 μl 1M Tris-HCl (In the refrigerator)

D) 100 μl 0.5M EDTA

2| AP Buffer

A) 40 ml nuclease-free water

B) 2.5 ml 20x SSC

C) 500 μl 10% SDS

3| GS Buffer (store at 65^o^C)

A) 49 ml nuclease-free water

B) 250 μl 20x SSC

C) 500 μl 10% SDS

M-280 Streptavidin coated Dynabead Preparation

1| Resuspend the Dynabeads M-280 Streptavidin by shaking the vial to obtain a homogeneous suspension.

2| Transfer 2.5 ml of Dynal beads to two 15 ml conical tube for a total volume of 5 ml (If doing fewer than 96 samples, for every sample to be captured, transfer 50 μl of Dynabeads to a 15 ml conical tube plus 4 additional samples worth of beads for dead volume and pipeting error).

3| Wash beads with 10 ml of BW buffer to each of the conical tubes containing the Dynal M-280 beads (For fewer than 96 samples, use 200 μl BW buffer per 50 μl beads).

4| Gently vortex and place tube in DynaMag-15 bead separator for 1-2 minutes. Remove the supernatant without disturbing the attached beads.

5| Repeat steps 3 and 4 two more times.

8| After the third buffer exchange, resuspend the DYNAl M-280 beads in 5 ml BW buffer (For fewer than 96 samples, use 100 μl BW buffer per 50 μl beads). Once the DYNAl M-280 have been resuspended, the content of each conical tube may be combined to ensure a homogenous mixture of washed M-280 beads.

9| Aliquot 102 μl of washed beads into each well of an Eppendorf 96 well twin.tec plate (beads location should match that of the sample location in the Hybridization plate).

*NOTE: Incorrect concentration of M-280 Dynabeads may result in low Catch product yield.*

10| Store plate at 4^o^C until use.

Deck Preparation

1| Wipe down Agilent Bravo deck with 70% ethanol.

2| Turn on the 2 heating blocks to 65^o^C

3| Add a box of 180μl tips to deck positions 3.

4| Aliquot 2 ml GS Buffer per sample well into a Costar 96-Deep well plate. Place in deck position 9.

5| Aliquot 180 μl of AP buffer in each well of an Eppendorf 96-well twin.tec plate and place plate in deck position 4.

6| Place plate of 100 μl/well washed Dynabeads into deck position 7.

7| Place Dynal MPC-96S magnet plate at deck position 5.

8| Place 96 well Hybridization/Target plate deck position 8.

9| Place an empty Costar 96-deep well plate at deck position 6.


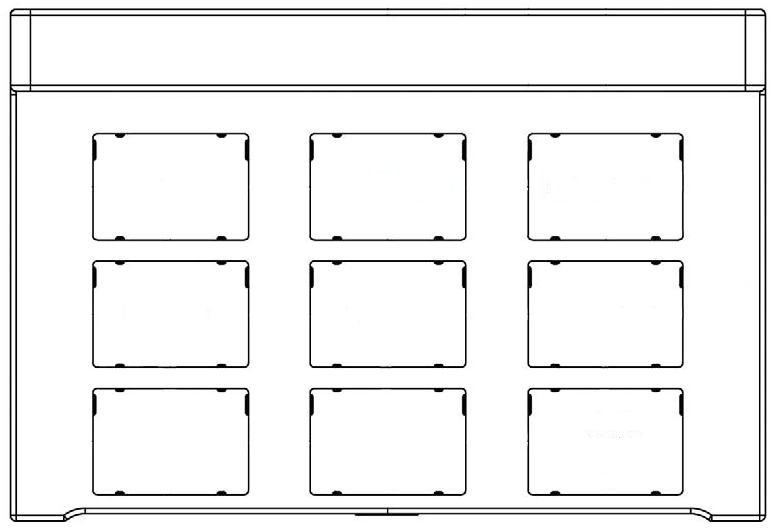


65°C Heat block

180ul Tips

AP Buffer Source plate

Dynal MPC-96s plate

Reagent Waste Costar 96-Deep Well plate

M-280 source plate / Catch Enrich. plate

Hyb./Target plate

GS Buffer Source plate on 65°C Heat block

! Caution: Immediately run the protocol when all reagents are in place.

Process Steps automated on the Bravo

*NOTE: Manual intervention is required ~ 3 hours and 45 minutes from the start of the Automated Capture protocol.*

**Sample Binding/Bead Addition**

1| Replace the used 180 μl tips from the previous step, with a box of 180 μl tips.

2| Place the Eppendorf twin.tec plate containing the washed Dynal M-280 beads at deck position 7.

3| Using a Dual height mix, ensure the M-280 beds are properly suspended. This may be achieved by setting setting the aspiration height to 1.45 mm from the bottom of the well and the dispense height at 11 mm from the bottom of the well. Mix approximately 5 times.

4| Aspirate 100 μl of washed M-280 beads from the M-280 source plate.

5| Dispense 100 μl of washed M-280 beads into the Hybridization/Target plate located at deck position 8.

6| Open Loop. Loop will be run 2 times.

7| Within the loop, perform a dual height mix of the M-280/sample mixture. Be sure to the aspiration height to 1.5 mm from the bottom of the well and the dispense height to 10 mm from the bottom of the well. Mix approximately 120 μl 100 times.

8| Close loop.

9| Place Hybridization/Target plate on Dynal MPC-96S magnet plate located at deck position 5. Let the Hybridization/Target plate to sit on the magnet plate for approximately 2 minutes. This will allow the M-280 streptavidin beads to separate from the Sample Binding (BW) buffer.

10| Remove supernatant and discard into the waste plate located at deck position 6.

11| Discard the used 180 μl tips.

**AP Buffer Wash**

1| Replace the used 180 μl tips from the previous step, with a box of clean 180 μl tips.

2| Open loop. This loop will be run 2 times.

3| Within loop, aspirate 180 μl of AP Buffer from the AP Buffer source plate located at deck position 4.

4| Still within loop, dispense the AP Buffer into the Hybridization/Target plate located at deck position 8.

5| Close loop.

6| Using a Dual Height Mix, mix approximately 120 μl in order to resuspend the M-280 beads in the AP buffer. This may be achieved by setting the aspiration height to 1.25 mm from the bottom of the well and the dispense height at 12 mm from the bottom of the well. Mix approximately 15 times.

7| Keeping the Hybridization/Target plate at deck position 8, allow the sample sit undisturbed for approximately 15 minutes.

8| Mix 120 μl of sample an additional 15 times.

9| Place Hybridization/Target plate on Dynal MPC-96S magnet plate located at deck position 5. Let the Hybridization/Target plate to sit on the magnet plate for approximately 2 minutes. This will allow the M-280 streptavidin beads to separate from the AP buffer.

10| Remove supernatant and discard into the waste plate located at deck position 6.

11| Discard the used 180 μl tips.

**GS Wash**

1| Replace the used 180 μl tips from the previous step, with a box of clean 180 μl tips.

2| Open loop. This loop will be run 2 times.

3| Within loop, aspirate 90 μl of GS Buffer from the GS Wash source plate located on a 65°C heat block at deck position 9.

4| Still within loop, Dispense the 180 μl heated GS buffer into the Hybridization/Target plate located at deck position 8.

6| Using a Dual Height Mix, mix approximately 120 μl in order to resuspend the M-280 beads in the GS wash buffer (Mix approximately 15 times). This may be achieved by setting the aspiration height to 1.25 mm from the bottom of the well and the dispense height at 12 mm from the bottom of the well. Mix approximately 15 times.

7| Place the Hybridization/Target plate on the 65°C heat block located at deck position 1.

8| Keeping the Hybridization/Target plate on the 65°C heat block, perform a Dual Height Mix. Mix 120 μl approximately 15 times. This may be achieved by setting the aspiration height to 1.25 mm from the bottom of the well and the dispense height at 11 mm from the bottom of the well. Mix approximately 15 times.

9| Allow the Hybridization/Target plate to sit on the heat block and incubate at 65°C for approximately 10 minutes.

10| Keeping the Hybridization/Target plate on the heat block, mix 75 μl of sample an additional 15 times. This may be achieved by setting the aspiration height to 1.25 mm from the bottom of the well and the dispense height at 8 mm from the bottom of the well. Mix approximately 15 times.

11| Place Hybridization/Target plate on Dynal MPC-96S magnet plate located at deck position 5. Let the Hybridization/Target plate to sit on the magnet plate for approximately 2 minutes. This will allow the M-280 streptavidin beads to separate from the GS Wash buffer.

12| Remove supernatant and discard into the waste plate located at deck position 6.

14| Discard the used 180 μl tips.

15| Repeat steps 1-14, five additional times.

*Critical Step: Prepare Mastermix on ice at step 10 of the 6^th^ GS Wash, or approximately 3 hours and 40 minutes from the start of the protocol. Store on ice until use.*

*Note: A user wait in the protocol will allow the user to replace the tip box in position 3 with a new 180 μl tip box for mastermix addition approximately 3.5 hours into the protocol.*

*Note: A user wait in the protocol will allow the user to replace the M-280 Streptavidin bead plate with a Twin Tec PCR 96 well plate containing 50 μl of PFU Mastermix in position 7 approximately 3.5 hours into the protocol.*

**Catch Enrichment PCR MasterMix addition**

*NOTE: It is recommended that the Catch Enrichment PCR MasterMix plate be on the deck as soon after the supernatant is removed during the final GS wash to avoid bead drying and static displacement.*

1| Thaw the reagents on ice.

2| Once the reagents have thawed, prepare the appropriate amount of mastermix for your samples plus and additional 10 samples for dead volume as detailed in Table 1.

3| Aliquot approximately 52 μl of Catch Enrichment mastermix to an Eppendorf 96-well twin.tec plate.

4| Once the final GS Wash has completed, replace the M-280 Streptavidin bead source plate located at deck position 7 with the Catch Enrichment mastermix plate.

5| Replace the used 180 μl tips from the previous step, with a box of clean 180 μl tips.

6| Aspirate 50 μl of the enrichment mastermix and dispense into the Hybridization/Target plate.

3| Perform a dual height mix, mixing approximately 45 μl twenty times.

4| Discard used 180 μl tips.

5| Using ABI Optical caps, seal Hybridization/Target plate containing samples.

6| Place in thermalcycler and begin Catch Enrichment PCR Program.

**Set up of the Agilent Bravo with LT head for Catch Enrichment 1.8 X Clean-up**

Catch Enrichment 1.8X Automated Clean up deck preparation

(Figure below displays deck layout)

1| Wipe down Agilent Bravo deck with 70% ethanol.

2| Add a box of 180μl tips to deck positions 1, 2 and 3.

3| Aliquot 90 μl of Agencourt AMPure XP beads in each well of a 96 well Eppendorf twin.tec plate, then place at deck position 8.

4| Aliquot 110 μl of 70% ethanol in each well of a 96 well Eppendorf plate, then place at deck position 5.

5| Aliquot 50 μl of 10 mM Tris-HC pH 8.0 in each well of a 96 well Eppendorf plate, the place at deck position 6.

6| Place the sample plate containing the catch enrichment PCR product on a Dynal MPC 96-S magnet plate, and place both the magnet plate and sample plate at deck position 4.

7| Place an unused Eppendorf 96 well twin.tec plate at position 7 and mark as enriched catch.

7| Place Dynal MPC 96S magnet plate at deck position 9.

**Process Steps automated on the Bravo**

*Note: Prior to starting the catch enrichment clean-up protocol, allow your sample plate to sit on the Dynal MPC 96-S magnet plate for approximately 2 minutes. This will allow the M-280 streptavidin beads to fully separate from your enrichment product.*

1| Put on clean 180 μl from tip box # 1.

2| Aspirate 55 μl of Catch Enrichment product from the sample plate, and dispense into the Eppendorf plate containing the AMPure XP beads located at deck position 8.

3| Perform a Dual Height Mix to ensure a homogenous mixture between the AMPure XP beads and the Catch enrichment product. Mix approximately 15 times.

4| Allow the sample plate to sit for 2 minutes, after which time place the sample plate, on a Dynal MPC – 96 S plate magnet for 4 minutes to allow the AMPure XP beads to separate from the solution.

5| Remove and discard the supernatant into the catch enrichment product plate located at deck position 4.

6| Discard the used 180 μl tips into tip box # 1.

7| Put on clean 180 μl tips from tip box # 2.

8| Leaving the sample plate on the Dynal MPC-96S magnet plate, aspirate 100 μl of 70% ethanol and dispense into the sample plate. DO NOT MIX.

9| Allow the AMPure XP beads and sample sit in the 70% ethanol for 30 seconds, then remove the ethanol and discard into the 70% ethanol source plate.

10| Discard tips into 180 μl tip box # 2.

11| Move the sample plate off of the Dynal MPC-96s magnet plate and allow the sample-AMPure bead complex to air dry for approximately 4 minutes at room temperature.

12| Put on clean 180 μl from tip box # 3.

13| Aspirate 40ml of Tris-HCl pH 8.0 from the Tris-HCl pH 8.0 source plate, and dispense into sample plate.

14| Pipette mix 15x and let stand at room temperature for approximately 2 minutes.

15| Place the sample plate, on a Dynal MPC – 96 S plate magnet for 3 minutes to allow the AMPure XP beads to separate from the solution.

16| Aspirate the eluate and dispense into the Eppendorf 96 well twin.tec plate located at position 7.

17| Discard tips into 180 μl tip box # 3.

18| Using optical caps, seal Eppendorf plate containing samples.

19| Proceed to sample quantification and normalization.


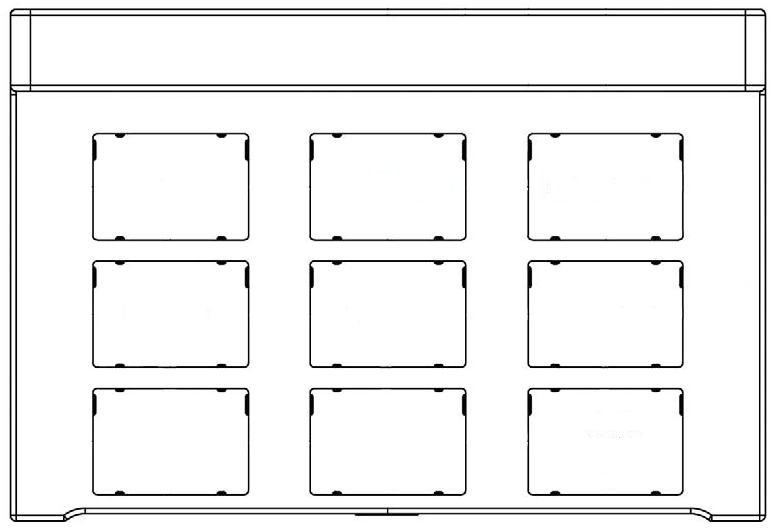


200ul Tips for binding and waste remove

200ul Tips for 70% ethanol Wash

200ul Tips for elution and final transfer

Catch Enrichment PCR Product

96-well Eppendorf PCR plate with 70% ethanol

96-well Eppendorf PCR plate with Elution Buffer

96-well Eppendorf PCR plate for final elute transfer

96-well Eppendorf PCR plate with AMPure XP beads

96-well PCR plate side magnet (Invitrogen)
